# Postovulatory ageing modifies sperm-induced Ca^2+^ oscillations in mouse oocytes through a conditions-dependent, multi-pathway mechanism

**DOI:** 10.1038/s41598-019-48281-3

**Published:** 2019-08-14

**Authors:** Marcin Szpila, Agnieszka Walewska, Dorota Sabat-Pośpiech, Patrycja Strączyńska, Takao Ishikawa, Robert Milewski, Katarzyna Szczepańska, Anna Ajduk

**Affiliations:** 1 Department of Embryology, Faculty of Biology, University of Warsaw, Miecznikowa 1, 02-096 Warsaw, Poland; 2 Department of Molecular Biology, Faculty of Biology, University of Warsaw, Miecznikowa 1, 02-096 Warsaw, Poland; 3 Department of Statistics and Medical Informatics, Medical University of Bialystok, Szpitalna 37, 15-295 Bialystok, Poland; 4 Present Address: Laboratory of RNA Biology and Functional Genomics, Institute of Biochemistry and Biophysics, Polish Academy of Sciences, Pawinskiego 5A, 02-106 Warsaw, Poland; 5 Present Address: Laboratory of Intracellular Ion Channels, Nencki Institute of Experimental Biology, Polish Academy of Sciences, Pasteura 3, 02-093 Warsaw, Poland; 6 Present Address: Cellular & Molecular Physiology, Institute of Translational Medicine, University of Liverpool, Crown St, Liverpool, L69 3BX UK; 7 Present Address: School of Medicine with the Division of Dentistry in Zabrze, Medical University of Silesia, pl. Traugutta 2, 41-800 Zabrze, Poland

**Keywords:** Ageing, Calcium signalling, Embryology

## Abstract

Postovulatory ageing of mammalian oocytes occurs between their ovulation and fertilization and has been shown to decrease their developmental capabilities. Aged oocytes display numerous abnormalities, including altered Ca^2+^ signalling. Fertilization-induced Ca^2+^ oscillations are essential for activation of the embryonic development, therefore maintaining proper Ca^2+^ homeostasis is crucial for the oocyte quality. In the present paper, we show that the mechanism underlying age-dependent alterations in the pattern of sperm-triggered Ca^2+^ oscillations is more complex and multifaceted than previously believed. Using time-lapse imaging accompanied by immunostaining and molecular analyses, we found that postovulatory ageing affects the amount of Ca^2+^ stored in the cell, expression of Ca^2+^ pump SERCA2, amount of available ATP and distribution of endoplasmic reticulum and mitochondria in a manner often strongly depending on ageing conditions (*in vitro* vs. *in vivo*). Importantly, those changes do not have to be caused by oxidative stress, usually linked with the ageing process, as they occur even if the amount of reactive oxygen species remains low. Instead, our results suggest that aberrations in Ca^2+^ signalling may be a synergistic result of ageing-related alterations of the cell cycle, cytoskeleton, and mitochondrial functionality.

## Introduction

Postovulatory ageing in mammals occurs between ovulation of mature oocytes and their fertilization and has been shown to decrease oocytes’ ability to react properly to sperm penetration and to develop further into the embryo^1–8^. Aged oocytes display a wide range of various abnormalities, such as dysfunction of actomyosin and microtubular cytoskeleton^8–13^, decreased activity of M-phase promoting kinases^11,14,15^, deregulation of energy metabolism^2,16–18^ and epigenetic alterations^19,20^. The extent of these abnormalities depends on the length of ageing period and, most likely, the genetic background of the cell. It has been also reported that postovulatory ageing disturbs Ca^2+^ signalling in oocytes and alters the pattern of sperm-induced Ca^2+^ oscillations^4,21–27^. The exact mechanism of this particular disturbance is not known, although it has been suggested that it may be caused by ageing-dependent oxidative stress^25^.

Fertilization-induced Ca^2+^ oscillations are essential for activation of the embryo development. They trigger processes such as completion of meiosis, establishment of the block to polyspermy and recruitment of maternal mRNAs required for activation of the embryonic genome^28,29^. In addition, Ca^2+^ oscillations regulate functioning of mitochondria^30–32^ and influence gene expression during further development of the embryo^33,34^. It has been shown that Ca^2+^ must be elevated for different time to induce properly each of these processes. If the number of Ca^2+^ transients is too low (*i*.*e*. the total duration of Ca^2+^ elevation is too short), the embryo implantation is impaired; on the other hand, an excessive number of Ca^2+^ transients (*i*.*e*. Ca^2+^ is elevated for too long) affects postimplantation development of the embryo^33–35^.

Ca^2+^ oscillations are triggered in oocytes by phospholipase C zeta (PLC zeta), introduced by a fertilizing sperm^36^. PLC zeta cleaves phosphatidylinositol 4,5-bisphosphate (PIP_2_) to 1,4,5-inositol triphosphate (IP_3_) and diacylglycerol. IP_3_ binds to its receptor (IP_3_ receptor type 1, IP3R1, dominates in oocytes^37^) located in the endoplasmic reticulum (ER) membrane. IP3R1 acts as a Ca^2+^ channel and opens upon IP_3_ binding, enabling the release of Ca^2+^ ions from the ER lumen into the cytoplasm. When the cytoplasmic Ca^2+^ concentration increases above a threshold level (low micromolar concentrations), the Ca^2+^ channel closes; the release of Ca^2+^ stops and the uptake of Ca^2+^ ions to the ER lumen is facilitated. The uptake of Ca^2+^ ions is mediated by ATP-dependent Ca^2+^ pumps (the sarco/endoplasmic reticulum Ca^2+^ ATPases, SERCA). Restoration of the Ca^2+^ level in the ER is required for the next Ca^2+^ release^38,39^. This interplay between IP3R1-mediated Ca^2+^ release and SERCA-mediated Ca^2+^ uptake constitutes the main mechanism of cytoplasmic Ca^2+^ oscillations observed in fertilized oocytes. Ca^2+^ oscillations cease after few hours when oocytes enter interphase^40–42^.

In the present paper, we show that postovulatory ageing in *in vivo* conditions has significantly more detrimental effect on Ca^2+^ homeostasis in mouse oocytes than *in vitro* ageing. In both conditions, postovulatory ageing affects several elements of the mechanism generating fertilization-induced Ca^2+^ oscillations, such as the amount of Ca^2+^ stored in the cell, expression of IP3R1 and SERCA2, the amount of available ATP and distribution of ER and mitochondria, but in a different way or to a different extent. We also argue that those changes are not necessarily caused by oxidative stress, as they occur even if the reactive oxygen species (ROS) level is not increased. Instead, our data suggest that aberrations in Ca^2+^ oscillatory pattern are a synergistic result of many ageing-related modifications, including alterations of the cell cycle, cytoskeleton, and mitochondrial functionality.

## Results and Discussion

### *In vitro* and *in vivo* postovulatory ageing differently alters the pattern of Ca^2+^ oscillations

**Figure 1:**
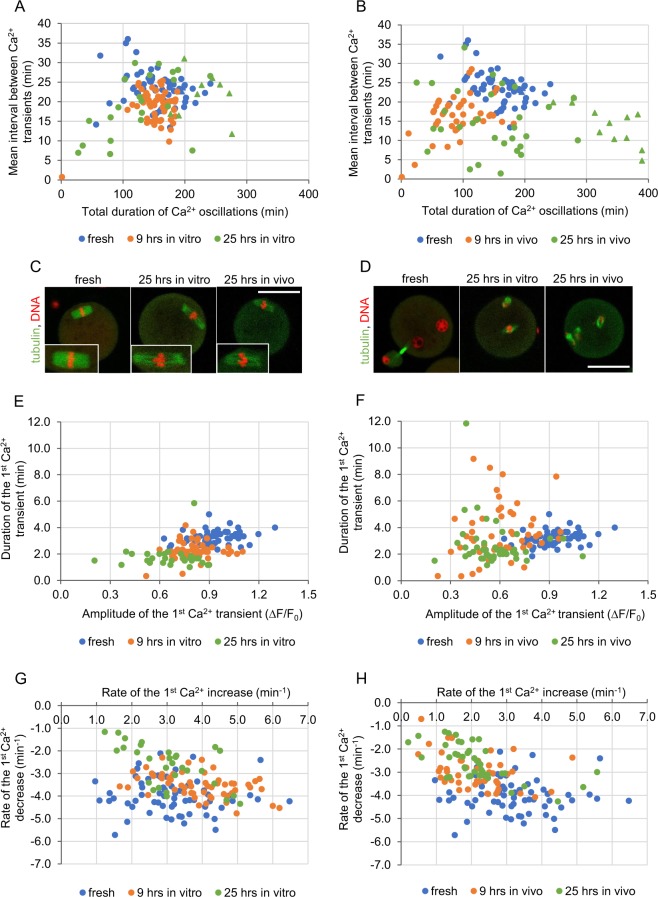
Fertilization-induced Ca^2+^ oscillations in postovulatory aged oocytes. **(A**,**B)** Point charts presenting the total duration of Ca^2+^ oscillations and the mean interval between Ca^2+^ transients in freshly ovulated oocytes, oocytes aged for 9 and 25 hrs *in vitro* **(A)** and *in vivo* **(B)**. **(C**,**D)** Representative immunostainings (β-tubulin in green, DNA in red) of nuclear apparatus in unfertilized freshly ovulated and aged oocytes **(C)**, and 6 hrs after fertilization of freshly ovulated and aged oocytes **(D)**. Scale bar 50 µm. **(E–F)** Point charts presenting the amplitude and the duration of the 1^st^ Ca^2+^ transient in freshly ovulated oocytes and oocytes aged for 9 and 25 hrs *in vitro* **(E)** and *in vivo* **(F)**. **(G–H)** Point charts presenting the rates of Ca^2+^ increase and decrease during the 1^st^ Ca^2+^ transient in freshly ovulated oocytes and oocytes aged for 9 and 25 hrs *in vitro* **(G)** and *in vivo* **(H)**. **(A,B, E–H)** Each dot/triangle represents one oocyte, the number of analysed oocytes is included in Table 1. Triangles (in **(A**,**B)**) mark oocytes that did not finish Ca^2+^ oscillations during the filming.

In order to examine the effect of different postovulatory ageing conditions (a short and a long duration, *in vitro* and *in vivo*) on the pattern of Ca^2+^ oscillations generated in fertilized mouse oocytes, we subjected mature, metaphase II oocytes to 9- or 25-hour-long aging, either in *in vitro* culture or in female oviducts. Then, we labelled oocytes for Ca^2+^ ions using a fluorescent dye Oregon Green 488 BAPTA-1AM (Oregon Green BAPTA), fertilized them *in vitro* and subjected to time-lapse imaging. Representative Ca^2+^ traces are presented in Supplementary Fig. S1. 9 hours of ageing, both in *in vitro* and *in vivo* conditions, increased the frequency of Ca^2+^ oscillations, as compared to freshly ovulated counterparts. However, only *in vivo* ageing shortened their total duration as well (Fig. 1A,B, Table 1). 25 hours of ageing led to more versatile reactions. In both *in vitro* and *in vivo* experimental variants, some oocytes displayed very frequent Ca^2+^ oscillations, while others generated Ca^2+^ transients with a frequency similar to that in freshly ovulated oocytes (on average, the mean interval between Ca^2+^ transients in *in vivo* aged oocytes was significantly shorter than in fresh ones). The same rule applied to the total length of Ca^2+^ oscillations: in some aged oocytes they were shorter and in some significantly longer than in freshly ovulated ones (Fig. 1A,B, Table 1). Although the average duration of Ca^2+^ oscillations in oocytes aged for 25 hrs did not differ from that observed in the fresh counterparts, in over 25% of aged oocytes Ca^2+^ oscillations were still in progress when the recording ended (even though they started on average at the same time after the imaging onset as in the fresh oocytes), suggesting that the 25-hour-long ageing may prolong Ca^2+^ oscillations. It has been shown that in mouse Ca^2+^ oscillations cease when fertilized oocytes enter interphase of the 1^st^ embryonic division. This decline may be caused by PLC zeta sequestration into the newly formed pronuclei^40–42^ (although this process seems not to be universal across species^43^) or/and decrease in IP3R1 phosphorylation by M-phase kinases, IP3R1 degradation, and change in ER distribution^44–47^. Oocytes aged for 25 hrs often did not form pronuclei during 7 hrs of the recording (data not shown), and instead they remained arrested in M-phase with disarrayed spindles, and, in consequence, displayed most likely high activity of M-phase kinases. It has been reported before that oocytes arrested in metaphase II by microtubule depolymerizing drugs, such as nocodazole or colcemid, generated significantly prolonged Ca^2+^ oscillations (lasting through the whole recording period, *i*.*e*. even up to 22 hrs) of low frequency and constant amplitude^48,49^. It seems that the inability of oocytes aged for 25 hrs to enter interphase is related to the spindle abnormality present already at the time of fertilization that inhibits M-phase/interphase transition through the spindle assembly checkpoint (SAC)^50^ (Fig. 1C,D). It has been shown previously that postovulatory ageing negatively affects microtubular cytoskeleton and spindle structure in oocytes^9,10^. Difficulties with the pronuclear formation in oocytes aged *in vitro* for 24 hrs have been also reported by Lacham-Kaplan and Trounson^3^.

**Table 1:** Effect of postovulatory aging on the pattern of Ca^2+^ oscillations and Ca^2+^ ER store in oocytes.

	fresh	9 hrs *in vitro*	25 hrs *in vitro*	9 hrs *in vivo*	25 hrs *in vivo*
	Median (Q1; Q3)				
No. of Ca^2+^ transients	**7**.**0**^a^ (6.0; 8.0) *n* = 64	**8**.**0**^f^ (7.0; 9.0) *n* = 57	**8**.**0** (6.0; 10.0) *n* = 30	**6**.**0**^f,b^ (5.0; 7.5) *n* = 51	**12**.**0**^a,b^ (7.0; 19.0) *n* = 43
Duration of Ca^2+^ oscillations (min)	**158**.**0**^a^ (128.3; 182.2) *n* = 64	**159**.**9**^b^ (141.7; 171.8) *n* = 57	**165**.**3**^c^ (107,1; 209.0) *n* = 30	**88**.**0**^a,b,c,d^ (64.1; 114.0) *n* = 47	**183**.**5**^d^ (117.9; 279.5) *n* = 42
Mean interval between Ca^2+^ transients during 1^st^ 2 hrs (min)	**23**.**4**^**a**,b,f^ (20.5; 25.3) *n* = 64	**19**.**6**^f^ (16.2; 21.3) *n* = 57	**20**.**7** (15.5; 26.0) *n* = 30	**16**.**8**^a^ (14.6; 19.0) *n* = 47	**14** ^b^ (10.1; 19.9) *n* = 42
Amplitude of the 1^st^ Ca^2+^ transient (ΔF/F_0_)	**0**.**9**^a,b,c^ (0.8; 1.0) *n* = 64	**0**.**8**^d,e^ (0.7; 0.9) *n* = 57	**0**.**7**^a^ (0.6; 0.8) *n* = 30	**0**.**6**^b,d^ (0.5; 0.7) *n* = 50	**0**.**6**^c,e^ (0.5; 0.6) *n* = 43
Amplitude of the 3^rd^ Ca^2+^ transient (ΔF/F_0_)	**0**.**8**^a,b,c^ (0.8; 0.9) *n* = 64	**0**.**8**^d,h^ (0.7; 0.8) *n* = 57	**0**.**7**^a,h^ (0.6; 0.7) *n* = 30	**0**.**7**^b^ (0.6; 0.7) *n* = 45	**0**.**6**^c,d^ (0.5; 0.6) *n* = 41
Duration of the 1^st^ Ca^2+^ transient (min)	**3**.**2**^a,b,f^ (2.8; 3.5) *n* = 64	**2**.**2**^a,g^ (2.0; 2.5) *n* = 57	**1**.**7**^b,c,h^ (1.4; 2.0) *n* = 30	**3**.**3**^c,g^ (2.5; 4.7) *n* = 50	**2**.**2**^f,h^ (1.8; 3.0) *n* = 43
Duration of the 3^rd^ Ca^2+^ transient (min)	**1**.**1** (1.0; 1.2) *n* = 64	**1**.**2** (1.0; 1.2) *n* = 57	**1**.**0** (0.8; 1.2) *n* = 30	**1**.**2** (1.0; 1.2) *n* = 45	**1**.**0** (0.8; 1.2) *n* = 41
Rate of the 1^st^ Ca^2+^ increase (min^−1^)	**3**.**1**^a,b^ (2.4; 3.7) *n* = 64	**3**.**9**^c,d^ (3.1; 4.6) *n* = 57	**3**.**0**^h,f^ (2.3; 3.5) *n* = 30	**2**.**2**^a,c,h^ (1.4; 2.7) *n* = 49	**2**.**0**^b,d,f^ (1.6; 2.3) *n* = 43
Rate of the 3^rd^ Ca^2+^ increase (min^−1^)	**4**.**7**^a,b,c^ (4.4; 5.2) *n* = 64	**4**.**3**^d,h^ (4.0; 4.6) *n* = 57	**3**.**7**^a,h^ (3.4; 4.0) *n* = 30	**3**.**8**^b^ (3.3; 4.2) *n* = 45	**3**.**1**^c,d^ (2.9; 3.3) *n* = 41
Rate of the 1^st^ Ca^2+^ decrease (min^−1^)	**−4**.**1**^a,b,c^ (**−**4.4; **−**3.6) *n* = 64	**−3**.**5**^d,f^ (**−**3.9; **−**3.2) *n* = 57	**−2**.**4**^a,f^ (**−**3.4; **−**2.0) *n* = 30	**−3**.**1**^b^ (**−**3.4; **−**2.5) *n* = 49	**−2**.**7**^c,d^ (**−**3.1; **−**2.1) *n* = 43
Rate of the 3^rd^ Ca^2+^ decrease (min^−1^)	**−4**.**7**^a,b,c^ (**−**5.2; **−**4.4) *n* = 64	**−4**.**2**^f,h^ (**−**4.5; **−**3.9) *n* = 57	**−3**.**4**^a,h^ (**−**3.8; **−**2.9) *n* = 30	**−3**.**9**^b^ (**−**4.2; **−**3.4) *n* = 45	**−3**.**2**^c,f^ (**−**3.7; **−**2.9) *n* = 41
Amplitude of the TG**-**induced Ca^2+^ release (ΔF/F_0_)	**1**.**6**^a,b,c,^ (1.4; 1.7) *n* = 65	**1**.**3**^a,f^ (1.3; 1.4) *n* = 75	**1**.**3**^b,g^ (1.2; 1.4) *n* = 53	**1**.**3**^c,f,g^ (1.2; 1.3) *n* = 63	**1**.**4**^c^ (1.3; 1.4) *n* = 56

Values marked with the same letter are significantly different: ^a,b,c,d,e^p < 0.001, ^f,g^p < 0.01, ^h^p < 0.05.

Postovulatory ageing *in vivo* decreased the amplitude of Ca^2+^ oscillations (we analysed in detail the 1^st^ and the 3^rd^ Ca^2+^ transient), both in the 9- and the 25-hour-long variants. In case of *in vitro* ageing, Ca^2+^ transients had lower amplitudes only after 25 hrs. We also noticed that the duration of the 1^st^ Ca^2+^ peak in oocytes aged for 9 hrs *in vitro*, but not *in vivo*, was shorter than in freshly ovulated ones, whereas in oocytes aged for 25 hrs the shortening occurred in both conditions (Fig. 1E,F, Table 1, Supplementary Fig. S2A,B). Ageing altered also dynamics of Ca^2+^ transients. In case of *in vitro* ageing (but only after 25 hrs), the concentration of cytoplasmic Ca^2+^ increased at a slower rate during the 1^st^ Ca^2+^ transient and decreased at a slower rate during the 1^st^ and the 3^rd^ Ca^2+^ transients while compared to fresh oocytes. In *in vivo* conditions, the increase and decrease of Ca^2+^ cytoplasmic levels during the 1^st^ and the 3^rd^ Ca^2+^ spikes slowed down already after 9 hrs and remained like this after 25 hrs (Fig. 1G,H, Table 1, Supplementary Figs S1B, S2C,D). Noteworthy, changes in the dynamics of Ca^2+^ oscillations observed in oocytes aged for 25 hrs (apart from the duration of Ca^2+^ oscillations in the *in vivo* group and the duration of the 1^st^ Ca^2+^ transient in the *in vitro* group) do not seem to be related to their M-phase kinase activity: they are comparable in oocytes that progressed to interphase after fertilization and in those that remained in M-phase (Supplementary Table S1). It is important to note that our measurements of the amplitudes and, in consequence, also the rates of Ca^2+^ increase/decrease, are only estimations, as Oregon Green BAPTA is not a ratiometric dye and its response to increasing Ca^2+^ concentration is not linear (its output flattens for higher Ca^2+^ concentrations)^51^. Nevertheless, the tendencies we observed in the analysed groups seem to be real, as they accord with the previously reported data^4,17,21,22,25^. Due to the characteristics of Oregon Green BAPTA susceptibility to Ca^2+^, the actual differences in the Ca^2+^ concentrations between the groups may be just more pronounced than what we have shown.

In summary, *in vivo* postovulatory ageing alters the Ca^2+^ oscillation pattern faster than *in vitro* ageing: it changed 8 out of 11 analysed parameters after 9 hrs, comparing to 2/11 in the *in vitro* variant. After 25 hrs in both variants more than half of the parameters were changed: 9/11 in *in vivo* and 6/11 in *in vitro* ageing. Ageing affected the frequency of Ca^2+^ transients, their amplitude, duration and dynamics of Ca^2+^ increase/decrease, and most likely, the total duration of Ca^2+^ oscillations. Interestingly, *in vivo* and *in vitro* aging affects differently not only Ca^2+^ oscillations, but also activities of M-phase kinases (MPF and MAPK) and profile of protein synthesis; in this case, the same as in our experiments, the effect of *in vivo* ageing is much more severe than that of *in vitro* aging^15,52^.

### Postovulatory ageing decreases the ER Ca^2+^ store and SERCA2 expression

**Figure 2:**
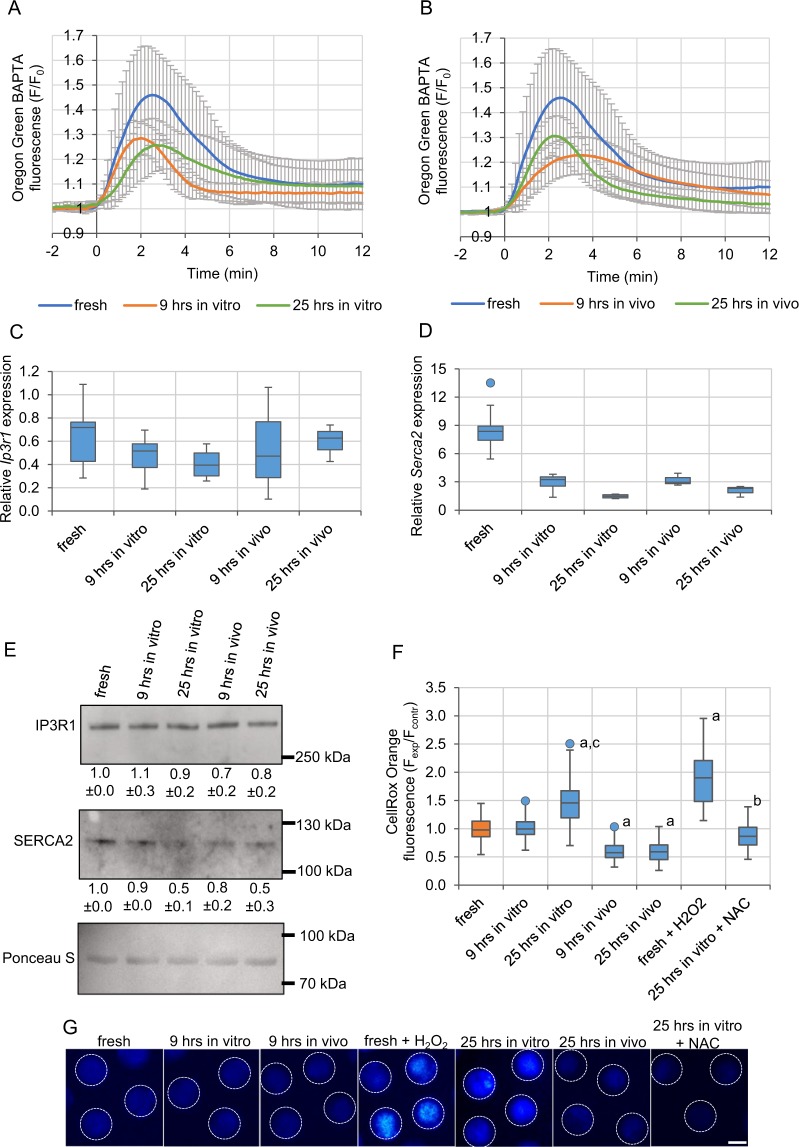
Effect of postovulatory ageing on Ca^2+^ ER store, expression of IP3R1 and SERCA2 and amount of ROS. **(A,B)** Mean Ca^2+^ release triggered by thapsigargin (TG), calculated for freshly ovulated oocytes and oocytes aged for 9 and 25 hrs *in vitro* **(A)** and *in vivo* **(B)**. The number of analysed oocytes is included in Table 1. Mean values +/− SD are shown. Time-point “0” was set as a moment when the cytoplasmic Ca^2+^ concentration in oocytes started to rise. **(C-D)** Relative expression of mRNA for *Ip3r1* (*Itpr1*) **(C)** and *Serca2* (*Atp2a2*) **(D)** genes in freshly ovulated and aged oocytes. **(E)** Western blot analysis of protein expression for IP3R1 and SERCA2. Results of the densitometric analysis are presented as numbers below the blots. Ponceau S staining was used to confirm an equal sample loading. **(F)** Mean intensity of CellROX Orange staining of reactive oxygen species (ROS) calculated for 65 freshly ovulated oocytes, 65 and 85 oocytes aged *in vitro* (for 9 and 25 hrs, respectively), 85 and 83 oocytes aged *in vivo* (for 9 and 25 hrs, respectively), 23 fresh oocytes treated with H_2_O_2_ and 28 oocytes aged for 25 hrs *in vitro* in medium supplemented with NAC. All intensity values were normalized with the mean fluorescence intensity calculated in the particular experiment for the control, freshly ovulated oocytes. ^a^p < 0.001 *vs*. fresh oocytes, ^b^p < 0.001 *vs*. 25hrs *in vitro*, ^c^p < 0.001 *vs*. 9 hrs *in vitro*. **(G)** CellROX Orange staining in representative oocytes from the experimental variants analysed in **(F)**. The dashed white line marks oocytes’ circumferences. Scale bar 50 µm. **(C-D, F)** Graphs present medians and the 1^st^ and the 3^rd^ quartile values. The ends of the whiskers are set at 1.5*IQR above the third quartile and 1.5*IQR below the first quartile. Dots show the minimum and maximum values if they are outside the range (outliers).

Ageing-related changes in the Ca^2+^ oscillation pattern may be caused by the modified amount of Ca^2+^ stored in oocytes or/and altered expression of proteins involved in generation of the oscillations. To assess the amount of Ca^2+^ stored in the ER, we labelled oocytes with Oregon Green BAPTA and treated with thapsigargin (TG), a SERCA inhibitor, which induces a release of Ca^2+^ from the ER cisterns to the cytoplasm. Increase in the Oregon Green BAPTA fluorescence observed afterwards reflects the size of the ER Ca^2+^ store. Both *in vitro* and *in vivo* ageing, for 9 and 25 hrs, led to a decrease in the amount of Ca^2+^ available from the ER (Fig. 2A,B, Table 1). We also examined mRNA and protein levels of two main regulators of Ca^2+^ oscillations: IP3R1 and SERCA2. Postovulatory ageing decreased both mRNA and protein levels of SERCA2, although the decline in the protein level appeared only after 25 hrs. In case of IP3R1, both mRNA and protein expression remained relatively constant during postovulatory ageing (Fig. 2C–E, Supplementary Fig. S2E).

Importantly, the decreased expression of SERCA2 protein, especially in *in vivo* aged oocytes, correlates with the slower rate of Ca^2+^ decrease during Ca^2+^ transients. Depleted ER Ca^2+^ stores explain, on the other hand, the lower amplitude of Ca^2+^ transients in postovulatory aged oocytes, which accords with the previous reports^4,53,54^. It has been suggested that the decreased amount of Ca^2+^ stored in the ER of ageing oocytes may be caused by a Ca^2+^ leak resulting from expression of a truncated caspase 3-cleaved form of IP3R1^27,55,56^, likely triggered by increased expression of miR-98^57^. The low amplitude of Ca^2+^ oscillations can be also related to the deregulation of other posttranslational modifications of IP3R1. It has been shown that IP3R1 functionality, particularly its sensitivity to IP_3_, depends on the phosphorylation status of the receptor^44,58,59^. MPF and MAPK are key kinases phosphorylating IP3R1 and stimulating its action^44,58,60^. As postovulatory ageing leads to a decrease in their activity^11,14,15,20^, IP3R1 phosphorylation, and, in result, its functionality, is inhibited^27^. Decreased functionality of IP3R1 could also explain the slower Ca^2+^ release rate during Ca^2+^ transients. However, the slower rate of Ca^2+^ rise could be caused also simply by the lower cellular Ca^2+^ stores^4,53,54^. Moreover, it is possible that postovulatory ageing affects plasma membrane channels responsible for Ca^2+^ entry. It has been shown recently that mouse oocytes lacking both TRPM7 and Ca_V_3.2 channels stop oscillating prematurely^61^. Inhibition of Ca^2+^ oscillations, in terms of the peak number and amplitude, has been also observed in porcine oocytes with downregulated expression of ORAI1 or STIM1 proteins, the key elements in the store-operated Ca^2+^ entry (SOCE) pathway, and in porcine oocytes overexpressing ORAI1 (but not STIM1)^62,63^.

### Cumulus cells are not sufficient to trigger the *in vivo* ageing phenotype

It has been suggested that cumulus cells extrude factors that accelerate ageing of oocytes^14,64–66^. We noticed that indeed, Ca^2+^ oscillations in oocytes aged for 9 hrs *in vivo*, *i*.*e*. in the company of cumulus cells, were shorter, displayed less Ca^2+^ spikes, and the 1^st^ Ca^2+^ transient had on average lower amplitude and slower rate of Ca^2+^ release than oocytes aged for the same time *in vitro*. Those differences between the ageing conditions diminished or disappeared after 25 hrs. We, therefore, examined whether the presence of cumulus cells during the 9-hour-long *in vitro* culture enhanced the effect of ageing on Ca^2+^ oscillations. Interestingly, we did not notice such a tendency. Although the amount of Ca^2+^ stored in the ER was depleted in oocytes cultured with cumulus cells to the level typical for oocytes aged *in vivo* (Supplementary Fig. S3A, Table 1, Supplementary Table S2), Ca^2+^ oscillations in oocytes aged for 9 hrs *in vitro* in presence of cumulus cells resembled – regarding all the above-mentioned parameters - oscillations in the *in vitro*, not *in vivo*, aged oocytes (Supplementary Fig. S3B–G, Table 1, Supplementary Table S2). Thus, cumulus cells are not sufficient to generate changes in Ca^2+^ oscillations typical for *in vivo* ageing. In this respect, our results accord with the data reported by Takahashi *et al*.^4^, who have also shown no pronounced negative effect of cumulus cells on fertilization-induced Ca^2+^ response in *in vitro* aged oocytes. Nonetheless, our data suggest that there are some factors present in an oviduct and absent in *in vitro* culture that accelerate oocyte ageing.

### Oxidative stress is not requisite for the age-related modifications of Ca^2+^ oscillations

It has been suggested in the literature that ageing-related changes in oocyte physiology, including Ca^2+^ homeostasis, can be attributed to the negative impact of oxidative stress^67–69^. To investigate this hypothesis, we first analysed the amount of reactive oxygen species (ROS) in oocytes aged *in vitro* and *in vivo* for 9 or 25 hrs. Measurements of fluorescence intensity of CellROX Orange, a ROS-sensitive dye, indicated that 9 hrs of *in vitro* ageing did not affect the abundance of ROS in oocytes, and a significant increase in ROS amount was observed only after 25 hrs. In contrast, both 9 and 25 hrs of *in vivo* ageing led to a decrease in ROS level, as compared to freshly ovulated oocytes (Fig. 2F,G).

**Figure 3:**
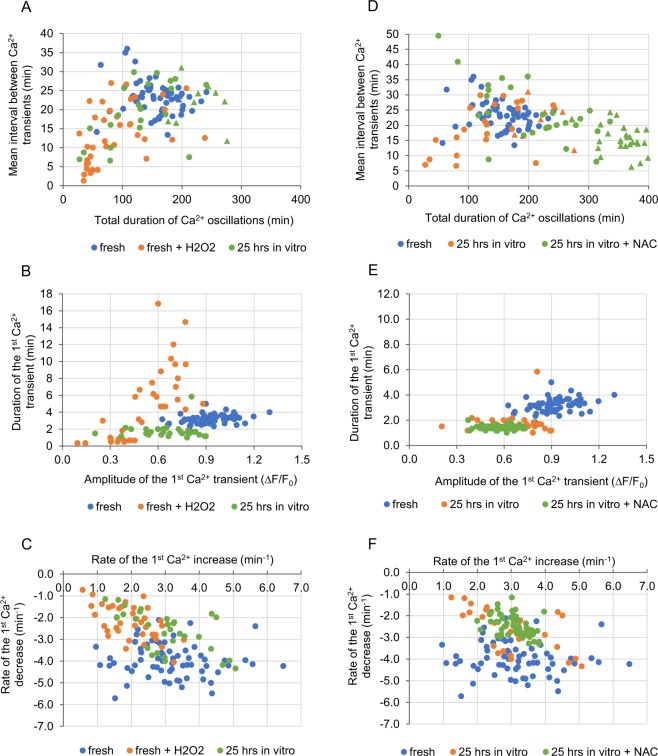
Impact of oxidative stress on Ca^2+^ homeostasis in oocytes. **(A–C)** Point charts presenting the total duration of Ca^2+^ oscillations and the mean interval between Ca^2+^ transients **(A)**, the amplitude and the duration of the 1^st^ Ca^2+^ transient **(B)** and the rates of Ca^2+^ increase and decrease during the 1^st^ Ca^2+^ transient **(C)** in fresh oocytes, fresh oocytes treated with H_2_O_2_ or oocytes aged for 25 hrs *in vitro*. **(D–F)** Point charts presenting the total duration of Ca^2+^ oscillations and the mean interval between Ca^2+^ transients **(D)**, the amplitude and the duration of the 1^st^ Ca^2+^ transient **(E)** and the rates of Ca^2+^ increase and decrease during the 1^st^ Ca^2+^ transient **(F)** in freshly ovulated oocytes and oocytes aged for 25 hrs *in vitro* with and without NAC. **(A–F)** Each dot/triangle represents one oocyte, the number of analysed oocytes is included in Tables 1 and 2. Triangles in **(A)** and **(D)** mark oocytes that did not finish Ca^2+^ oscillations during the filming.

Although oxidative stress did not seem to be related to altered Ca^2+^ homeostasis in *in vivo* aged oocytes or in oocytes aged *in vitro* for 9 hrs, it still may have been responsible for changes in Ca^2+^ signalling recorded in oocytes aged *in vitro* for 25 hrs. As 15 min incubation in 100 µM H_2_O_2_ induces a similar increase in the ROS level as 25-hour-long *in vitro* ageing (Fig. 2F,G), we examined whether it could also mimic the ageing impact on Ca^2+^ oscillations. Experiments with TG and A23187 ionophore showed that H_2_O_2_ led to severe depletion of the ER Ca^2+^ stores, but not to depletion of Ca^2+^ stored in other cellular compartments (Supplementary Fig. S4A). Moreover, oocytes pre-treated with H_2_O_2_ and then fertilized generated Ca^2+^ transients of lower amplitude and slower dynamics of Ca^2+^ increase/decrease than fresh untreated oocytes, the same as oocytes aged for 25 hrs *in vitro*. On the other hand, H_2_O_2_ treatment did not shorten the duration of the 1^st^ Ca^2+^ spike, as 25-hour-long *in vitro* ageing did. Instead, it shortened the total duration of Ca^2+^ oscillations, the mean interval between subsequent Ca^2+^ spikes and the duration of the 3^rd^ Ca^2+^ spike – alterations that were not recorded in oocytes aged *in vitro* for 25 hrs (Fig. 3A–C, Tables 1, 2, Supplementary Fig. S4B–D).

**Table 2:** Effect of oxidative stress, antioxidants, actin depolymerisation and parthenogenetic activation on the pattern of Ca^2+^ oscillations and Ca^2+^ ER store in oocytes.

	fresh + H_2_O_2_	25 hrs *in vitro* + NAC	fresh + CCD	fresh + EtOH
	Median (Q1; Q3)			
No. of Ca^2+^ transients	**8**.**0** (5.3; 10.0) *n* = 38	**14**.**0**^aaa,bb,cc,ddd^ (11.0; 19.8) *n* = 54	**9**.**5**^aaa,ddd^ (7.0; 11.0) *n* = 98	**7**.**0**^e^ (5.0; 12.0) *n* = 59
Duration of Ca^2+^ oscillations (min)	**71**.**4**^aaa,bbb,cc,eee^ (45.5; 113.9) *n* = 38	**315**.**6**^aaa,bbb,ccc,ddd,e^ (225.0; 360.9) *n* = 54	**196**.**1**^a,b,ddd^ (174.9; 217.8) *n* = 97	**105**.**8**^a,bb,ccc,e^ (86.5; 126.6) *n* = 59
Mean interval between Ca^2+^ transients during 1^st^ 2 hrs (min)	**12**.**9**^aaa, c^ (7.2; 19.3) *n* = 38	**18**.**8**^aa^ (14.4; 23.3) *n* = 54	**18**.**0**^aaa^ (13.8; 22.9) *n* = 97	**18**.**8**^aaa^ (10.1; 26.5) *n* = 59
Amplitude of the 1^st^ Ca^2+^ transient (ΔF/F_0_)	**0**.**5**^aaa,bbb^ (0.4; 0.7) *n* = 38	**0**.**6**^aaa,bbb^ (0.5; 0.6) *n* = 53	**0**.**9**^cc,ddd,eee^ (0.8; 1.0) *n* = 95	**0**.**6**^aaa,bbb^ (0.4; 0.7) *n* = 59
Amplitude of the 3^rd^ Ca^2+^ transient (ΔF/F_0_)	**0**.**5**^aaa,bbb,d^ (0.3; 0.6) *n* = 38	**0**.**6**^aaa,bbb,d^ (0.5; 0.6) *n* = 52	**0**.**9**^ccc,ddd,eee^ (0.8; 0.9) *n* = 95	**0**.**6**^aaa,bbb^ (0.5; 0.7) *n* = 59
Duration of the 1^st^ Ca^2+^ transient (min)	**4**.**7**^ccc^ (0.7; 7.0) *n* = 37	**1**.**3**^aaa,bbb,ddd,eee^ (1.3; 1.5) *n* = 53	**3**.**3**^bbb,ccc,ee^ (3.0; 3.7) *n* = 94	**2**.**8**^ccc^ (2.3; 3.5) *n* = 59
Duration of the 3^rd^ Ca^2+^ transient (min)	**1**.**3**^a,cc,eee^ (1.2; 1.6) *n* = 38	**1**.**0** (0.8; 1.2) *n* = 52	**1**.**0** (0.8; 1.2) *n* = 38	**1**.**0**^b^ (0.7; 1.2) *n* = 59
Rate of the 1^st^ Ca^2+^ increase (min^−1^)	**2**.**0**^aaa,bbb,cc^ (1.5; 2.4) *n* = 38	**2**.**5**^bbb^ (2.1; 2.8) *n* = 53	**2**.**9**^bb,ddd,eee^ (2.3; 3.4) *n* = 87	**1**.**8**^aaa,bbb,ccc^ (1.3; 2.4) *n* = 58
Rate of the 3^rd^ Ca^2+^ increase (min^−1^)	**2**.**7**^aaa,bbb,dd^ (1.4; 3.5) *n* = 36	**3**.**1**^aaa,bbb,d^ (2.7; 3.4) *n* = 52	**4**.**7**^ccc,ddd,eee^ (4.2; 5.2) *n* = 91	**3**.**3**^aaa,bbb^ (2.6; 4.0) *n* = 59
Rate of the 1^st^ Ca^2+^ decrease (min^−1^)	**−2**.**2**^aaa,bbb,d^ (**−**2.8; **−**1.6) *n* = 38	**−2**.**0**^aaa,bbb,ddd^ (**−**2.3; **−**1.7) *n* = 53	**−4**.**1**^ccc,ddd,eee^ (**−**4.5; **−**3.6) *n* = 87	**−3**.**0**^aaa,b^ (**−**3.6; **−**2.2) *n* = 58
Rate of the 3^rd^ Ca^2+^ decrease (min^−1^)	**−2**.**8**^aaa,bbb,dd^ (**−**3.4; **−**1.7) *n* = 36	**−2**.**5**^aaa,bbb,ddd,e^ (**−**2.8; **−**2.1) *n* = 52	**−4**.**8**^ccc,ddd,eee^ (**−**5.3; **−**4.3) *n* = 91	**−3**.**5**^aaa,b^ (**−**4.1; **−**2.8) *n* = 59
Amplitude of the TG**-**induced Ca^2+^ release (ΔF/F_0_)	n/a	**1**.**3**^a,bbb,ccc,eee^ (1.2; 1.3) *n* = 42	n/a	n/a

^aaa^p < 0.001, ^aa^p < 0.01, ^a^p < 0.05 ***vs***. **fresh oocytes**, ^bbb^p < 0.001, ^bb^p < 0.01, ^b^p < 0.05 ***vs***. **9** **h** ***in vitro***,

^ccc^p < 0.001, ^cc^p < 0.01, ^c^p < 0.05 ***vs***. **25** **h** ***in vitro***, ^ddd^p < 0.001, ^dd^p < 0.01, ^d^p < 0.05 ***vs***. **9** **h** ***in vivo***, ^eee^p < 0.001, ^ee^p < 0.01, ^e^p < 0.05 ***vs***. **25** **h** ***in vivo***.

We also investigated whether changes in Ca^2+^ homeostasis caused by 25-hour-long *in vitro* ageing could be rescued with antioxidant treatment. To this end, oocytes were cultured for 25 hrs in medium supplemented with 5 mM *N*-acetylcysteine (NAC)^70^. The amount of ROS in such oocytes was significantly lower than in oocytes aged without NAC and similar to the ROS level in fresh oocytes (Fig. 2F,G). Interestingly, this decrease in the ROS amount prevented neither the age-dependent decrease in the ER Ca^2+^ store (Supplementary Fig. S4E, Table 2) nor the change in the pattern of Ca^2+^ oscillations. The amplitudes of Ca^2+^ transients, the duration of the 1^st^ Ca^2+^ spike and the rates of increases and decreases in Ca^2+^ during Ca^2+^ spikes were the same in oocytes aged with and without NAC. Moreover, Ca^2+^ oscillations lasted for even longer and were even more frequent in oocytes aged with NAC than in those aged in a pure medium (Fig. 3D–F, Tables 1, 2, Supplementary Fig. S4F–H).

Taken together, our data indicate that oxidative stress may not be responsible for the alterations in Ca^2+^ homeostasis observed in postovulatory aged oocytes. First, the increase in ROS was not observed in the majority of our ageing variants, even though the Ca^2+^ oscillations were disturbed in all of them. Secondly, although some of the changes in Ca^2+^ oscillations recorded in oocytes treated with H_2_O_2_ resembled those in aged ones, this resemblance may be at least partially incidental, as treatment with the antioxidant did not reverse the ageing phenotype.

### Ageing affects the functionality of mitochondria

**Figure 4:**
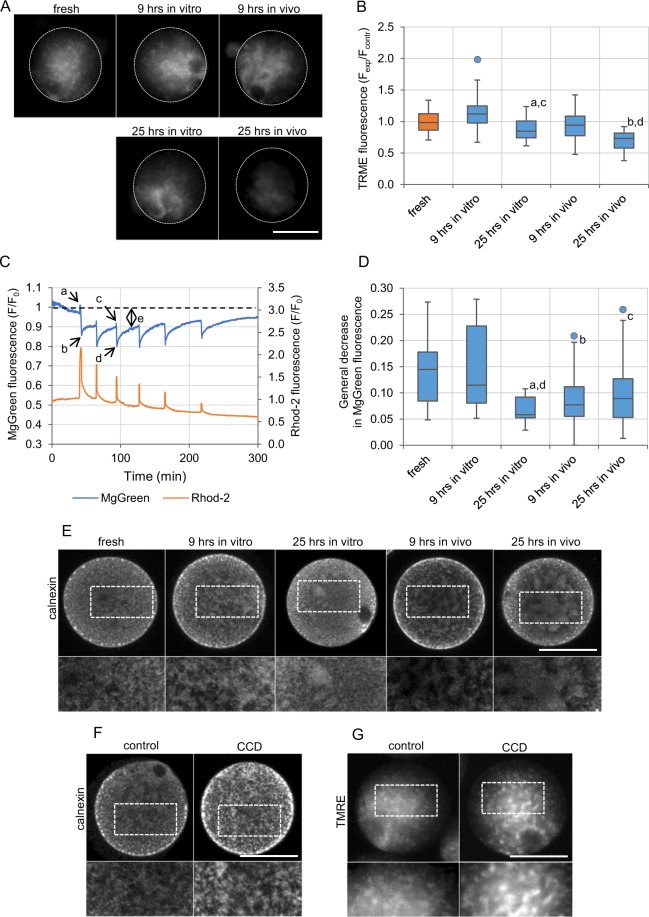
Functionality of mitochondria and distribution of organelles in postovulatory aged oocytes. **(A)** TMRE staining of active mitochondria in representative freshly ovulated oocytes and oocytes aged *in vitro* and *in vivo* for 9 and 25 hrs. The dashed white line marks oocytes’ circumferences. Scale bar 50 µm. **(B)** Mean intensity of TMRE staining (indicative of mitochondrial membrane potential) for the experimental variants depicted in **(A)** calculated for 43 freshly ovulated oocytes, 47 and 44 oocytes aged *in vitro* (for 9 and 25 hrs, respectively) and 58 and 53 oocytes aged *in vivo* (for 9 and 25 hrs, respectively). All intensity values were normalized with the mean fluorescence intensity calculated in the particular experiment for the control, freshly ovulated oocytes. ^a^p < 0.05, ^b^p < 0.001 *vs*. fresh oocytes, ^c^p < 0.001 *vs*. 9 hrs *in vitro*, ^d^p < 0.001 *vs*. 9 hrs *in vivo*. **(C)** Ca^2+^ (in orange; Rhod-2 fluorescence) and Mg^2+^ (in blue; MgGreen fluorescence) oscillations in a representative freshly ovulated oocyte. The cytoplasmic concentration of free Mg^2+^ ions is inversely proportional to the ATP concentration. Letters a-e indicate values used for further analysis of the fertilization-induced ATP production and presented in the graph **(D)** and Supplementary Fig. S6E,F. (**D)** General decrease in the Mg^2+^ concentration (value ‘e’ in **(C)**), indicative of general increase in the ATP production, calculated for 31 freshly ovulated oocytes, 24 and 10 oocytes aged *in vitro* (for 9 and 25 hrs, respectively), and 17 and 30 oocytes aged *in vivo* (for 9 and 25 hrs, respectively). ^a^p < 0.01, ^b^p < 0.05, ^c^p = 0.05 *vs*. fresh oocytes, ^d^p < 0.05 *vs*. 9 hrs *in vitro*. **(B,D)** Graphs present medians and the 1^st^ and the 3^rd^ quartile values. The ends of the whiskers are set at 1.5*IQR above the third quartile and 1.5*IQR below the first quartile. Dots show the minimum and maximum values if they are outside the range (outliers). **(E-F)** Immunofluorescence staining of calnexin, an ER marker, in representative freshly ovulated oocytes and oocytes aged *in vitro* and *in vivo* for 9 and 25 hrs **(E)**, and in control and cytochalasin D (CCD)-treated oocytes **(F)**. The dashed white line marks the zoomed regions. Scale bar 50 µm. **(G)** TMRE staining of active mitochondria in representative control and CCD-treated oocytes. The dashed white line marks the zoomed regions. Scale bar 50 µm.

Since oxidative stress does not seem to be the main cause of defective Ca^2+^ homeostasis in postovulatory aged oocytes, we wished to investigate other potential mechanisms. The activity of mitochondria and Ca^2+^ oscillations are intertwined^71,72^, so disrupted functionality of these organelles may be a reason for altered Ca^2+^ homeostasis in postovulatory aged oocytes. To investigate this possibility, we examined how postovulatory ageing affected the functionality of mitochondria. To this end, we used TMRE, a fluorescent probe of mitochondrial membrane potential^51^. TMRE staining revealed that 25-hour-long ageing, both *in vitro* and *in vivo*, caused severe aggregation of active mitochondria (Fig. 4A). Additionally, we also observed a decrease in the TMRE fluorescence intensity in those two experimental groups (Fig. 4B). Even though TMRE has certain limitations as a potentiometric dye^73^, it suggests that the prolonged ageing leads to a decrease in the mitochondrial membrane potential.

Next, we wished to examine whether postovulatory ageing impaired Ca^2+^-induced ATP production, typical for fertilized oocytes. To this end we labelled oocytes with fluorescent dyes: Magnesium Green (MgGreen) for Mg^2+^ and Rhod-2 for Ca^2+^ ions (concentration of Mg^2+^ reflects inversely ATP level, i.e. it decreases when ATP concentration increases and *vice versa*^74^; see Methods section), fertilized them and subjected to time-lapse imaging. The specificity of MgGreen dye towards Mg^2+^ *vs*. Ca^2+^ ions was tested by challenging the labelled oocytes with FCCP or TG. As expected, FCCP led to an abrupt increase in the MgGreen fluorescence reflecting a decrease in the ATP amount caused by uncoupling oxidation from phosphorylation in mitochondria^75^, whereas TG did not alter significantly the MgGreen fluorescence (Supplementary Fig. S5A,B). We also did not observe MgGreen sequestration into the ER cisterns (Supplementary Fig. S5C).

As shown before^2^, in fresh oocytes fertilization induced oscillatory changes in the MgGreen fluorescence reflecting readjustments of the ATP concentration during the sperm-triggered Ca^2+^ transients. Those oscillations were accompanied by a general decrease in the MgGreen fluorescence, indicative of general increase in the ATP production observed after fertilization (Fig. 4C,D). In *in vivo* aged (for 9 and 25 hrs) oocytes, the general decrease in the MgGreen signal was lower than in freshly ovulated oocytes. A similar decrease in the ATP production was also observed in oocytes aged *in vitro* for 25 hrs (Fig. 4D, Supplementary Fig. S6A–D). Additionally, aged oocytes (25 hrs *in vitro* and 9 and 25 hrs *in vivo*) displayed difficulties in readjusting ATP levels after fertilization-induced Ca^2+^ transients (Supplementary Fig. S6E,F): a decrease in the MgGreen fluorescence (i.e. increase in the ATP level) during the 1^st^ and the 3^rd^ Ca^2+^ transients was significantly lower than in freshly ovulated oocytes. Therefore, it seems that ageing may decrease the functionality of mitochondria that in consequence may affect ATP-dependent generation of Ca^2+^ transients and explain some of the alterations in the pattern of Ca^2+^ oscillations. This is, however, a two-way process, as hindering Ca^2+^ oscillations may further affect the ATP production^31,32^. Interestingly, despite the fact that mitochondrial dysfunction is often linked to oxidative stress^76,77^ (we noticed that it accompanies the alleviation of ROS concentration in oocytes aged *in vitro* for 25 hrs), we observed it also in oocytes aged *in vivo*, in which ROS levels were actually decreased in comparison to the fresh oocytes.

### Changes in actin cytoskeleton and organelle distribution modify the pattern of Ca^2+^ oscillations

**Figure 5:**
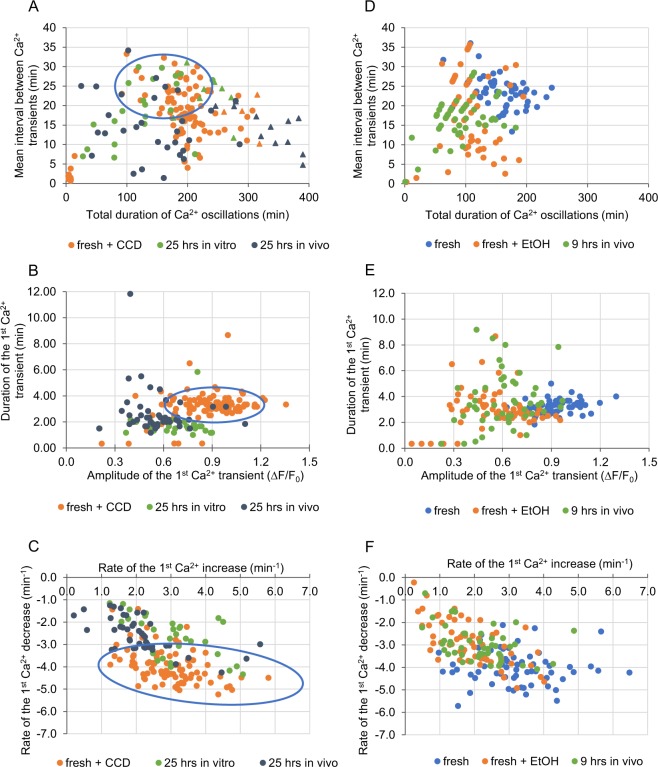
Impact of actin depolymerisation and parthenogenetic activation on Ca^2+^ homeostasis. **(A**–**C)** Point charts presenting the total duration of Ca^2+^ oscillations and the mean interval between Ca^2+^ transients **(A)**, the amplitude and the duration of the 1^st^ Ca^2+^ transient **(B)** and the rates of Ca^2+^ increase and decrease during the 1^st^ Ca^2+^ transient **(C)** in freshly ovulated oocytes treated with CCD, and oocytes aged for 25 hrs *in vitro* and *in vivo*. Blue ovals indicate the region, where dots representing freshly ovulated oocytes would have been located. **(D-F)** Point charts presenting the total duration of Ca^2+^ oscillations and the mean interval between Ca^2+^ transients **(D)**, the amplitude and the duration of the 1^st^ Ca^2+^ transient **(E)** and the rates of Ca^2+^ increase and decrease during the 1^st^ Ca^2+^ transient **(F)** in freshly ovulated oocytes, oocytes pre-activated parthenogenetically with EtOH and oocytes aged for 9 hrs *in vivo*. Each dot/triangle represents one oocyte, the number of analysed oocytes is included in Tables 1 and 2. Triangles (in **(A)** and **(D)**) mark oocytes that did not finish Ca^2+^ oscillations during the filming.

Postovulatory ageing often leads to aggregation of organelles, including mitochondria and ER cisterns^10,17,18,78^. Indeed, we showed that ageing alters the structure of ER and mitochondria network, facilitating formation of the cistern aggregates (Fig. 4A,E). As ER and mitochondria localization depends on actin filaments^79,80^, such redistribution may be related to a decreased functionality of actin cytoskeleton observed in aged oocytes^12,13^. Therefore, we wished to examine whether dysfunctional actin cytoskeleton and, in consequence, redistribution of ER and mitochondrial network, altered Ca^2+^ signalling. To hinder actin cytoskeleton and trigger aggregation of organelles, we treated fresh oocytes with cytochalasin D (CCD), an actin-depolymerizing agent. As expected, we observed aggregates of ER cisterns and mitochondria similar to those in postovulatory aged oocytes, although never as pronounced as in oocytes aged for 25 hrs *in vivo* (Fig. 4F,G). In fertilized oocytes, CCD treatment did not change the characteristics of the single Ca^2+^ transients, but it altered the pattern of Ca^2+^ oscillations as a whole: increased the duration and the frequency of oscillations as well as the number of Ca^2+^ transients. Similar trends were observed in postovulatory aged oocytes, especially in 25-hour-long variants (Fig. 5A–C, Tables 1, 2, Supplementary Fig. S7A–C). Indeed, the data published previously indicate that generation of proper Ca^2+^ oscillations in response to fertilization depends on distribution of the ER cisterns^46^^,^^79,81^. Interestingly, it has been also shown in somatic cells that intact cortical actin cytoskeleton is required for maintaining ER-plasma membrane junctions that participate in Ca^2+^ signalling and lipid (including phosphatidylinositol) metabolism^82,83^. Moreover, it seems that actin cytoskeleton regulates also ER-mitochondria contact sites that are crucial for Ca^2+^ signalling in the cell^84–86^.

### Premature activation modifies the pattern of Ca^2+^ oscillations

Last but not least, altered Ca^2+^ homeostasis in postovulatory aged oocytes may be caused by deregulation of the cell cycle. As we described before, prolonged Ca^2+^ oscillations in oocytes aged for 25 hrs are likely related to the inability of those oocytes to enter the 1^st^ embryonic interphase. In oocytes aged for 9 hrs *in vivo*, we observed another cell cycle abnormality: they tend to undergo spontaneous activation before fertilization, which was manifested as formation of the 2^nd^ polar bodies (Supplementary Fig. S8A). It has been reported that postovulatory ageing decreases the activity of M-phase kinases in mouse oocytes, which in turn could induce such a phenotype^11,14,15,20,67^. To test whether modifications of Ca^2+^ oscillations typical for 9-hour-long *in vivo* ageing were related to this phenomenon, we analysed the pattern of Ca^2+^ oscillations in oocytes pre-treated with ethanol (EtOH), a parthenogenetic agent. EtOH induces in oocytes a single large Ca^2+^ transient^87^. It activated fresh oocytes very effectively, so within approx. 1.5 hrs they reached the same stage of activation as seen in oocytes aged for 9 hrs *in vivo* (Supplementary Fig. S8A). Consequently, it is likely that the activity of M-phase kinases important for regulation of the Ca^2+^ signalling (see previous paragraphs) decreased in the EtOH-treated oocytes to the same level as in the aged oocytes. We showed that in the pre-activated oocytes the duration of Ca^2+^ oscillations, the frequency and the amplitude of Ca^2+^ transients, as well as the rate of Ca^2+^ increase/decrease during the transients were similar to those in oocytes aged for 9 hrs *in vivo* and significantly decreased as compared to the fresh oocytes. Moreover, parameters such as the duration of the 1^st^ and the 3^rd^ Ca^2+^ transients or the number of Ca^2+^ transients remained unchanged compared to fresh oocytes, the same as in oocytes aged for 9 hrs *in vivo* (Fig. 5D–F, Tables 1, 2, Supplementary Fig. S8B–D).

As ageing-related premature activation of oocytes does not necessarily involve an increase in the cytoplasmic Ca^2+^ concentration, we decided to examine additionally how Ca^2+^-independent parthenogenetic activation affected the pattern of Ca^2+^ oscillations. To this end, we incubated fresh oocytes with 6-DMAP, an inhibitor of protein kinases, and cycloheximide (CHX), an inhibitor of protein synthesis. This procedure activated oocytes less uniformly and efficiently than EtOH and at the time of fertilization, usually only a subset of cells formed anaphase bulges (as opposed to the 2^nd^ polar bodies visible in the majority of EtOH-treated oocytes and the oocytes aged for 9 hrs *in vivo*) (Supplementary Fig. S8A). 6-DMAP/CHX treatment seemed to have a more diverse effect on the pattern of Ca^2+^ oscillations in oocytes than EtOH pre-activation or 9-hour-long *in vivo* ageing: e.g. in some oocytes the duration of Ca^2+^ oscillations or the rates of Ca^2+^ rise/decline were lower than in fresh oocytes, whereas in others such decrease did not occur. Nevertheless, on average its impact on Ca^2+^ oscillations resembled in many ways that of the above-mentioned experimental conditions: it decreased the duration of Ca^2+^ oscillations and the rate of Ca^2+^ increase/decrease during the transients, and did not affect the duration of the 3^rd^ Ca^2+^ transient and the number of Ca^2+^ spikes. On the other hand, it did not increase the frequency nor decrease the amplitudes of Ca^2+^ transients (Supplementary Table S2, Supplementary Fig. S9).

Alterations in the pattern of Ca^2+^ oscillations caused by EtOH and 6-DMAP/CHX treatments varied slightly, possibly due to a different extent of the cell cycle progression (*i*.*e*. decrease in the activity of M-phase kinases) or to the different mechanism of activation. Ca^2+^-induced activation leads for example to degradation of IP3R1 receptor^88^ that most likely is not observed in Ca^2+^-independent mode of oocyte stimulation. Taken together, these results suggest, however, that spontaneous activation may be at least partially responsible for the modification of the Ca^2+^ oscillation pattern in *in vivo* aged oocytes.

### The pattern of Ca^2+^ oscillations and embryo fragmentation

**Figure 6:**
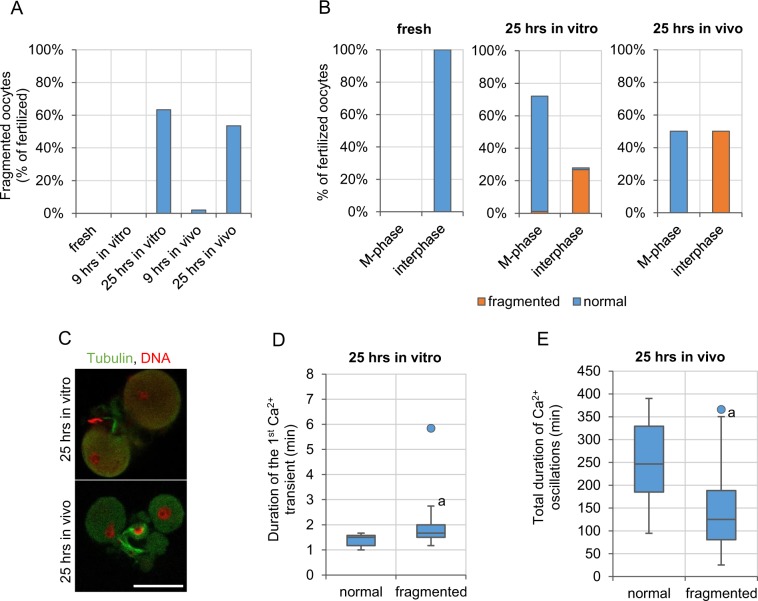
Developmental potential of postovulatory aged oocytes. **(A)** Frequency of fragmentation in fertilized freshly ovulated oocytes (n = 64) and oocytes aged for 9 and 25 hrs *in vitro* and *in vivo* (n = 57 and 30 and n = 51 and 43, respectively). **(B)** Percentage of oocytes arrested in metaphase II or activated (in interphase) among fertilized freshly ovulated oocytes and oocytes aged for 9 and 25 hrs *in vitro* and *in vivo*, and frequency, with which they undergo fragmentation. **(C)** Immunostaining (β-tubulin in green, DNA in red) or representative fragmented embryos derived from oocytes aged for 25 hrs *in vitro* and *in vivo*. Scale bar 50 µm. **(D)** The duration of the 1^st^ Ca^2+^ transient in normal and fragmented embryos derived from oocytes aged for 25 hrs *in vitro*. **(E)** The total duration of Ca^2+^ oscillations in normal and fragmented embryos derived from oocytes aged for 25 hrs *in vivo*. **(D**,**E)** Graphs present medians and the 1^st^ and the 3^rd^ quartile values. The ends of the whiskers are set at 1.5*IQR above the third quartile and 1.5*IQR below the first quartile. Dots show the minimum and maximum values if they are outside the range (outliers). ^a^p < 0.01 *vs*. normal, non-fragmented embryos.

It has been suggested that change in the pattern of Ca^2+^ oscillations caused by postovulatory ageing may lead to oocyte apoptosis and fragmentation^23,24,89^. Pro-apoptotic stimuli provided by postovulatory ageing, such as the decreased amount of BCL-2, ATP or increased oxidative stress^4,6,10,17,24,90,91^, may push aged oocytes upon fertilization to cell death. Indeed, majority of oocytes aged for 25 hrs (63.3% (19/30) in *in vitro* and 53.5% (23/43) in *in vivo* conditions) underwent fragmentation (believed to be usually an effect of apoptosis^92,93^) within few hours after fertilization, while fragmentation occurred neither in freshly ovulated oocytes, nor oocytes aged for 9 hrs (Fig. 6A). Interestingly, fragmentation in aged oocytes was observed only in those activated (*i*.*e*. in oocytes that completed meiosis and entered the 1^st^ embryonic interphase); fertilized oocytes that had remained arrested in metaphase II did not undergo this process (Figs 1D, 6B,C). Therefore, we analysed which alterations in the pattern of Ca^2+^ oscillations correlate with this age-related susceptibility to fragmentation. In *in vitro* aged oocytes, fragmentation was more likely if the 1^st^ Ca^2+^ transient lasted for longer (p < 0.01, Fig. 6D). It seems plausible, that too long increase in cytoplasmic Ca^2+^ concentration translates to Ca^2+^ overload in mitochondria, where it can cause a persistent increase in permeability of the mitochondrial membrane, and in result, trigger release of proapoptotic factors, such as cytochrome *c*^94^. On the other hand, in *in vivo* aged group, fragmentation correlated with the shorter total duration of Ca^2+^ oscillations (p < 0.001, Fig. 6E). It could be related to the fact that short Ca^2+^ oscillations reflect the depleted Ca^2+^ ER store, which in turn may lead to ER stress and activate the unfolded protein response that triggers cell death^95^. A similar relationship has been observed before, but for *in vitro* aged oocytes^23,24^.

In summary, we show that the effect of postovulatory ageing on Ca^2+^ homeostasis in oocytes is much more complex and multifaceted than it has been reported before. Our results indicate that in order to understand it properly many various factors have to be taken into consideration, oxidative stress, often associated with age-related aberrations, being only one of them. Ageing-induced alterations in mitochondrial and cytoskeletal functionality, organelle distribution and cell cycle regulation, seem to be of special importance here. As postovulatory ageing affects oocytes of all mammalian species studied so far, including livestock and humans^5,9,10,17,18,78^, further research on this process has a practical meaning, as it may help to optimize *in vitro* fertilization procedure.

Moreover, our observations lead to an exciting question regarding the mechanism, in which the oviductal environment accelerates oocyte ageing. It seems plausible that the accelerated deterioration of oocytes in *in vivo* conditions, preventing their development after fertilization, is evolutionarily justified. Embryonic development in mammals is very costly for a female organism and it may be beneficial to enable it only when fertilization occurs on time, ascertaining good quality of the fertilizing sperm. Indeed, it has been reported that in humans probability of successful conception is the highest when insemination takes place just before or on the day of ovulation^96^.

## Methods

### Animals and reagents

F1 (C57Bl6/Tar × CBA/Tar) mice were maintained in the Animal Facility of the Faculty of Biology, University of Warsaw at 14:10 light/dark cycle and provided with food and water *ad libitum*. Animals were sacrificed by cervical dislocation. All experiments were approved by the Local Ethics Committee for Experimentation on Animals no. 1 (Warsaw, Poland) and were performed in compliance with the national regulations. All reagents were purchased from Sigma-Aldrich unless stated otherwise.

### Oocyte collection

Female mice were superovulated with an intraperitoneal injection of 10 IU of pregnant mare serum gonadotrophin (PMSG, Intervet) followed 48 hrs later by 10 IU of human chorionic gonadotrophin (hCG, Intervet). Ovulated oocytes were recovered from oviducts: (i) 15 hrs after hCG (fresh and *in vitro* aged oocytes); (ii) 24 hrs after hCG (oocytes aged *in vivo* for 9 hrs); (iii) 40 hrs after hCG (oocytes aged *in vivo* for 25 hrs). Except for some experiments (ageing with cumulus cells), recovered oocytes were placed in hyaluronidase solution (150 IU/ml in PBS) to remove the cumulus cells. Denuded oocytes were washed in M2 medium (M16 buffered with Hepes^97^) and either subjected to further experimental procedures or cultured *in vitro* for 9 or 25 hrs (*in vitro* ageing for 9 and 25 hrs) in M2 medium and 37.5 °C and 5% CO_2_ in the air. In some experiments, freshly ovulated oocytes were incubated for 4 hrs with or without cytochalasin D (CCD; 2 µg/ml in M2 medium). Some fresh oocytes were also parthenogenetically activated with 8% ethanol solution (in M2 medium, for 8 min) or with 2.5 mM 6-DMAP and 10 μg/ml cycloheximide (CHX) (in M2 medium for the whole period of preparatory manipulations (isolation, staining, etc.) *i*.*e*. approx. 1.5 hrs), and others – treated for 15 min with 100 µM H_2_O_2_ (in M2 medium). In some experiments, oocytes were aged *in vitro* for 25 hrs in M2 medium supplemented with 5 mM *N*-acetylcysteine^70^.

### Sperm collection

Epididymal sperm was isolated from male mice and capacitated in 0.5 ml of fertilization medium with 5 mg/ml BSA^98^ for 1.5–2 hrs in 37.5 °C and 5% CO_2_ in the air.

### Imaging of Ca^2+^ oscillations

Oocytes loaded with 5 μM fluorescent Ca^2+^ indicator Oregon Green 488 BAPTA-1AM (Oregon Green BAPTA; Molecular Probes, Thermo Fisher Scientific; in pure M2 medium or M2 medium supplemented with 2 µg/ml CCD or with 2.5 mM 6-DMAP and 10 μg/ml CHX, 30 min) were subjected to acidic Tyrode’s solution (pH 2.5)^99^ in order to remove *zonae pellucidae*. Oocytes without *zonae* were transferred to a glass-bottom dish (MatTek Corporations) with M2 medium without BSA and allowed to stick to the glass bottom (in some experiments M2 medium without BSA but with 2 µg/ml CCD was used). Next, the dish was placed on the time-lapse imaging system (Zeiss Axiovert microscope with AxioCam HRm camera) equipped with an environmental chamber sustaining a temperature of 37.5 °C. 1-2 μl of capacitated sperm suspension was added to the oocytes. The experiment was repeated 3-5 times for each variant with consistent results (Supplementary Dataset 1).

Time-lapse imaging with single-plane images taken every 10 s was performed for approx. 7 hrs. Oocytes were illuminated with light passing through a 450–490 nm excitation filter, and the emitted light was collected with a 500–550 nm emission filter (exposure time 50 ms, 4 × 4 binning). Changes in cytoplasmic Ca^2+^ concentration were assessed by measuring the mean intensity of Oregon Green BAPTA fluorescence in time. To avoid any additional variability between the experiments caused by the different extent of dye loading, the initial (pre-fertilization) mean intensity of fluorescence was calculated for each oocyte and then used to normalize the measurements in this oocyte. The resulting values are ratios: measured fluorescence intensity (F) /initial fluorescence intensity (F_0_). The rates of increase and decrease of Ca^2+^ concentration during the Ca^2+^ transients were calculated as tangents of the rising/decreasing slopes of the Ca^2+^ transients (‘a’ parameter in y = ax + b linear function fitted into these slopes; Supplementary Fig. S1B).

### Ca^2+^ store measurement

Oocytes were loaded with 5 μM Oregon Green BAPTA (in M2 medium, 30 min) and transferred to a glass-bottom dish with M2 medium devoid of Ca^2+^ and Mg^2+^ ions. After 2 minutes of time-lapse imaging (conditions the same as described in ‘Imaging of Ca^2+^ oscillations’ section) thapsigargin (TG) or A23187 ionophore were added to the medium (to the final concentration of 10 μM and 1 μM, respectively). Single-plane images were taken every 10 s for approximately 30 min (the settings were the same as described for imaging of Ca^2+^ oscillations). Changes in the Oregon Green BAPTA fluorescence were analysed as described for the imaging of Ca^2+^ oscillations.

### Assessment of ATP level

To assess changes in the ATP concentration during fertilization, we visualised Mg^2+^ ions, as described by Igarashi *et al*.^2^. The intracellular Mg^2+^ pool is predominantly present as MgATP^2−^ and the affinity of ATP^4−^ for Mg^2+^ is about 10-fold greater than that of ADP at physiological pH. Therefore, Mg^2+^ increases when ATP hydrolysis exceeds the rephosphorylation of ADP to ATP and *vice versa*^74^. To simultaneously image changes in the cytoplasmic Ca^2+^ and Mg^2+^ concentrations, oocytes were incubated for 30 min with 5 μM Rhod-2 AM (Molecular Probes, Thermo Fisher Scientific) and 5 μM Magnesium Green AM (MgGreen; Molecular Probes, Thermo Fisher Scientific), respectively. Then, their *zonae pellucidae* were removed with acidic Tyrode’s solution and oocytes were transferred to a glass-bottom dish with M2 medium without BSA and allowed to stick to the glass bottom. Next, they were placed on the time-lapse imaging system (Zeiss Axiovert microscope with AxioCam HRm camera) equipped with an environmental chamber sustaining a temperature of 37.5 °C, and fertilized as described in ‘Imaging of Ca^2+^ oscillations’ section. To test the specificity of MgGreen towards Mg^2+^ ions *vs*. Ca^2+^ ions, oocytes, labelled as described above, were subjected in separate experiments to 1 μM FCCP or 10 μM TG while imaged. Imaging was performed in normal M2 medium or M2 medium devoid of Ca^2+^ and Mg^2+^ ions, respectively.

Time-lapse imaging with single-plane images taken every 10 s was performed for approx. 7 hrs for the fertilization experiments and 30 min for the experiments testing MgGreen specificity. Oocytes were illuminated with light passing through 450–490 nm and 538–562 nm excitation filters, and the emitted light was collected with 500–550 nm and 570–640 nm emission filters, for MgGreen and Rhod-2, respectively (exposure times 20 and 50 ms, 4 × 4 binning). Changes in the cytoplasmic Ca^2+^ concentration were assessed by measuring the mean intensity of Rhod-2 fluorescence in time, whereas changes in the Mg^2+^ concentration – the mean intensity of MgGreen fluorescence. To avoid any additional variability between the experiments caused by the different extent of dye loading, the initial (pre-fertilization) mean intensity of fluorescence was calculated for each oocyte and then used to normalize the measurements in this oocyte. The resulting values were ratios: measured fluorescence intensity (F) /initial fluorescence intensity (F_0_). Additionally, a drift caused by the photobleaching of MgGreen was calculated for each experiment (we used traces obtained for unfertilized oocytes) and then subtracted from the experimental measurements for fertilized oocytes.

### Visualisation of reactive oxygen species

Oocytes were loaded with 5 µM CellROX Orange (Molecular Probes, Thermo Fisher Scientific; in M2 medium, 30 min) and transferred to a glass-bottom dish with M2 medium. Single equatorial plane images of the oocytes were taken on a fluorescence microscope (Zeiss Axiovert microscope with AxioCam HRm camera) equipped with an environmental chamber sustaining a temperature of 37.5 °C. Oocytes were illuminated with light passing through a 538–562 nm excitation filter, and the emitted light was collected with a 570–640 nm emission filter (exposure time 100 ms, 4 × 4 binning). ROS concentration was assessed by measuring the mean intensity of CellROX Orange fluorescence. In each experiment the mean fluorescence intensity in aged/treated oocytes (F_exp_) was normalized with the mean fluorescence intensity in control, freshly ovulated oocytes dyed and imaged simultaneously (F_contr_).

### Visualization of mitochondria

Oocytes were loaded with 100 nM TMRE (in M2 medium) for 30 min and transferred to a glass-bottom dish with M2 medium. Single equatorial plane images of the oocytes were taken on a fluorescence microscope (Zeiss Axiovert microscope with AxioCam HRm camera) equipped with an environmental chamber sustaining a temperature of 37.5 °C. Oocytes were illuminated with light passing through a 538–562 nm excitation filter, and the emitted light was collected with 570–640 nm emission filter (exposure time 50 ms, 1 × 1 binning). As TMRE is a potential-sensitive mitochondrial dye^51^, we assessed the mitochondrial membrane potential in oocytes by measuring the mean intensity of TMRE fluorescence. In each experiment the mean fluorescence intensity in aged/treated oocytes (F_exp_) was normalized with the mean fluorescence intensity in control, freshly ovulated oocytes dyed and imaged simultaneously (F_contr_).

### Immunofluorescence staining

Oocytes were fixed in 4% PFA (30 min, RT), permeabilized with 0.5% Triton-X100 (30 min, RT) and blocked with 3% BSA. Calnexin, an ER marker, was labelled with a rabbit polyclonal antibody (1:200, Abcam) followed by an Alexa Fluor 633-conjugated goat anti-rabbit IgG (1:200; Invitrogen, Thermo Fisher Scientific) and β-tubulin – with a mouse monoclonal antibody conjugated with FITC (1:50). Embryos were incubated in the primary antibodies overnight at 4 °C, washed in PBS and 3% BSA and then, if required, incubated with the secondary antibody for 2 hrs in RT. DNA was stained with propidium iodide (0.01 mg/ml in PBS; 30 min, RT or overnight, 4 °C). Oocytes were analysed on an inverted confocal microscope (Zeiss and Olympus).

### Western blotting

Expression of IP3R1 and SERCA proteins was examined in samples of 50–80 oocytes, depending on the experiment. Cell lysates were mixed with 4× NuPage LDS sample Buffer and 10× NuPage Sample Reducing Agent (Invitrogen, Thermo Fisher Scientific) and heated for 10 min in 70 °C. The samples were subjected to NuPage Novex 3–8% Tris-Acetate gels (Invitrogen, Thermo Fisher Scientific) and separated proteins were transferred onto PVDF membranes (Hyperbond-P, Amersham Biosciences). The blots were then stained with Ponceau S to confirm the equal sample load, blocked in 5% non-fat milk in TTBS and probed for 1 h with a rabbit polyclonal antibody (Rbt03) raised against a 15 amino acid peptide sequence of the C-terminal end of the IP3R1^100^ and with a goat anti-SERCA2 antibody, diluted 1:350 and 1:200, respectively, in 5% non-fat milk in TTBS. A donkey anti-goat antibody and a goat anti-rabbit antibody (Bio-Rad), respectively, conjugated with horseradish peroxidase diluted 1:5000 were used as secondary antibodies in 1-hour-long incubation. Detection was performed by the enhanced chemiluminescence technique using SuperSignal West Dura Extended Duration Substrate reagents (Pierce, Thermo Fisher Scientific) according to the manufacturer’s instruction. Western blot analysis was repeated 3 times for IP3R1 and 2 times for SERCA2.

### Real-time RT-PCR

Oocytes were transferred in groups of 30 into 20 μl of Lysis/Binding Buffer (Dynabeads mRNA DIRECT Micro Kit, Thermo Fisher Scientific) and stored in −80 °C until further analysis. mRNA was isolated from the samples using the Dynabeads mRNA DIRECT Micro Kit (Thermo Fisher Scientific) according to the manufacturer’s protocol. In short, thawed samples were rotated with 10 μl of paramagnetic oligo-(dT)_25_ bead suspension for 30 min at RT. mRNA was eluted from the beads by adding 10 μl of DEPC-treated water and heated for 10 min at 70 °C with 0.5 μg oligo-(dT)_25_. The reverse transcription was performed in a total volume of 20 μl using 200 U of Superscript II Reverse Transcriptase, 0.5 mM dNTPs and 40 IU RNase inhibitor (Invitrogen, Thermo Fisher Scientific) at 42 °C for 50 min. Synthesized cDNA was diluted twice with nuclease-free water (Thermo Fisher Scientific) and subjected to preamplification procedure (T100 thermocycler, Bio-Rad; 10 cycles: 95 °C/15 s, 60 °C/4 min) with TaqMan PreAmp Mix (Thermo Fisher) and pooled TaqMan Gene Expression Assay probes (*Itpr1*/IP3R1: cat. no. Mm00439907_m1; *Atp2a2*/SERCA2: Mm01275320_m1; *Actb*/Actin B: Mm01205647_g1; Applied Biosystems, Thermo Fisher Scientific). The preamplification product was diluted twice and subjected to a real-time PCR using TaqMan Gene Expression MasterMix in StepOne Real-Time PCR System thermocycler (Applied Biosystems, Thermo Fisher Scientific; 50 °C/2 min; 60 °C/10 min; 50 cycles: 95 °C/15 sec, 60 °C/1 min). The relative level of expression was calculated using 2^−ΔCt^ method^101^ with actin B expression used for normalization. PCR analysis was repeated 3–9 times depending on the experimental variant.

### Statistical analysis

Kruskal-Wallis test with the post-hoc analysis and Mann–Whitney U test were applied to analyse the data. The differences between groups were considered statistically significant for p < 0.05.

## Supplementary information

Supplementary Information

Supplementary Dataset 1
