# Supplementary material for: Postovulatory ageing modifies sperm-induced Ca2+ oscillations in mouse oocytes through a conditions-dependent, multi-pathway mechanism
Source: Sci Rep. 2019 Aug 14;9:11859. doi: 10.1038/s41598-019-48281-3 (PMC6694115; doi:10.1038/s41598-019-48281-3)

SUPPLEMENTARY INFORMATION

for

„Postovulatory ageing modifies sperm-induced Ca^{2+} oscillations in mouse oocytes through a conditions-dependent, multi-pathway mechanism”

Marcin Szpila, Agnieszka Walewska, Dorota Sabat-Pośpiech, Patrycja Strączyńska, Takao Ishikawa, Robert Milewski, Katarzyna Szczepańska and Anna Ajduk

Supplementary Table S1. Effect of the cell cycle status on the pattern of Ca^{2+} oscillations in oocytes aged for 25 hrs *in vitro* and *in vivo*

	25 hrs *in vitro*		25 hrs *in vivo*	
	M-phase-arrested	Progressing to interphase	M-phase-arrested	Progressing to interphase
	**Median** (Q1;Q3)			
No. of Ca^{2+} transients	**9.0**<br>(7.5; 10.5)<br>*n=11*	**7.0**<br>(6.0; 9.0)<br>*n=19*	**14.0**<br>(9.5; 24.3)<br>*n=20*	**9.0**<br>(5.5; 15)<br>*n=23*
Duration of Ca^{2+} oscillations (min)	**199.1**<br>(144.4; 242.9)<br>*n=11*	**130.2**<br>(97.9; 185.0)<br>*n=19*	**246.4**[b]<br>(185.2; 329.1)<br>*n=19*	**125.0**[b]<br>(80.7; 188.5)<br>*n=23*
Mean interval between Ca^{2+} transients during 1st 2 hrs (min)	**24.4**<br>(14.4; 26.3)<br>*n=11*	**18.8**<br>(15.6; 23.9)<br>*n=19*	**13.5**<br>(10.3; 19.2)<br>*n=19*	**14.5**<br>(9.8; 21.6)<br>*n=23*
Amplitude of the 1st Ca^{2+} transient ($\Delta F/F_0$)	**0.7**<br>(0.6; 0.8*)*<br>*n=11*	**0.7**<br>(0.5; 0.8)<br>*n=19*	**0.5**<br>(0.5; 0;7)<br>*n=20*	**0.6**<br>(0.5; 0.6)<br>*n=23*
Amplitude of the 3rd Ca^{2+} transient ($\Delta F/F_0$)	**0.7**<br>(0.6; 0.7)<br>*n=11*	**0.7**<br>(0.6; 0.7*)*<br>*n=19*	**0.6**<br>(0.5; 0.6)<br>*n=19*	**0.6**<br>(0.5; 0.7)<br>*n=22*
Duration of the 1st Ca^{2+} transient (min)	**1.5**[a]<br>(1.2; 1.6)<br>*n=11*	**1.7**[a]<br>(1.5; 2.0)<br>*n=19*	**2.2**<br>(2.0; 2.6)<br>*n=20*	**2.3**<br>(1.8; 3.3)<br>*n=23*
Duration of the 3rd Ca^{2+} transient (min)	**1.0**<br>(0.8; 1.0)<br>*n=11*	**1.0**<br>(1.0; 1.2)<br>*n=19*	**1.0**<br>(1.0; 1.2)<br>*n= 19*	**0.8**<br>(0.8; 1.0)<br>*n=22*
Rate of the 1st Ca^{2+} increase (min^{-1})	**3.4**<br>(2.8; 4.2)<br>*n=11*	**3.0**<br>(2.0; 3.2*)*<br>*n=19*	**2.1**<br>(1.4; 2.2)<br>*n=20*	**2.0**<br>(1.8; 2.4)<br>*n=23*
Rate of the 3rd Ca^{2+} increase (min^{-1})	**3.7**<br>(3.5; 4.0)<br>*n=11*	**3.7**<br>(3.2; 4.0)<br>*n=19*	**3.0**<br>(2.7; 3.4)<br>*n=19*	**3.1**<br>(3.1; 3.3)<br>*n=22*
Rate of the 1st Ca^{2+} decrease (min^{-1})	**-2.7**<br>(-3.1; -2.1)<br>*n=11*	**-2.3**<br>(-3.5; -1.8)<br>*n=19*	**-2.6**<br>(-3.1; -1.8)<br>*n=20*	**-2.8**<br>(-3.1; -2.1)<br>*n=23*
Rate of the 3rd Ca^{2+} decrease (min^{-1})	**-3.6**<br>(-3.7; -3.2)<br>*n=11*	**-3.3**<br>(-3.9; -2.8)<br>*n=19*	**-3.1**<br>(-3.8; -2.8)<br>*n=19*	**-3.3**<br>(-3.7; -3.0)<br>*n=22*

Values marked with the same letter are significantly different: [a,b]$p<0.01$,

Supplementary Table S2. Effect of cumulus cells and Ca^{2+}-independent parthenogenetic activation on the pattern of Ca^{2+} oscillations and the ER Ca^{2+} store in oocytes

	9 hrs *in vitro*<br>+ CC	fresh<br>+ 6-DMAP + CHX
	**Median** (Q1;Q3)	
No. of Ca^{2+} transients	**9.0**^{a,ddd}<br>(8.0; 10.0)<br>*n=67*	**6.0**^{bbb,c,eee}<br>(5.0; 7.0)<br>*n=62*
Duration of Ca^{2+} oscillations (min)	**146.1**^{ddd}<br>(135.9; 160.9)<br>*n=67*	**120.0**^{a,b,eee}<br>(63.2; 162.4)<br>*n=61*
Mean interval between Ca^{2+} transients during 1st 2 hrs (min)	**17.0**^{aaa}<br>(15.1; 19.5)<br>*n=67*	**21.1**^{d,eee}<br>(18.3; 24.0)<br>*n=61*
Amplitude of the 1st Ca^{2+} transient ($\Delta F/F_0$)	**0.8**^{ddd,eee}<br>(0.7; 0.9)<br>*n=67*	**1.2**^{bbb,ccc,ddd,eee}<br>(1.0; 1.3)<br>*n=60*
Amplitude of the 3rd Ca^{2+} transient ($\Delta F/F_0$)	**0.8**^{c,eee}<br>(0.7; 0.8)<br>*n=67*	**1.0**^{aa,bbb,ccc,ddd,eee}<br>(1.0; 1.1)<br>*n=56*
Duration of the 1st Ca^{2+} transient (min)	**2.7**^{ccc}<br>(2.2; 3.2)<br>*n=67*	**2.4**^{aaa,c,d}<br>(2.0; 2.7)<br>*n=60*
Duration of the 3rd Ca^{2+} transient (min)	**1.2**^{c,eee}<br>(1.2; 1.3)<br>*n=67*	**1.1**<br>(1.0; 1.3)<br>*n=56*
Rate of the 1st Ca^{2+} increase (min^{-1})	**3.4**^{ddd,eee}<br>(2.8; 4.2)<br>*n=67*	**2.4**^{bbb}<br>(1.5; 3.5)<br>*n=59*
Rate of the 3rd Ca^{2+} increase (min^{-1})	**4.3**^{eee}<br>(3.9; 4.6)<br>*n=67*	**3.4**^{aaa,e}<br>(2.8; 5.5)<br>*n=56*
Rate of the 1st Ca^{2+} decrease (min^{-1})	**-3.3**^{aa}<br>(-3.5; -2.9)<br>*n=67*	**-3.2**^{aa,e}<br>(-4.4; -2.5)<br>*n=60*
Rate of the 3rd Ca^{2+} decrease (min^{-1})	**-4.3**^{cc,eee}<br>(-4.5; -3.8)<br>*n=67*	**-3.5**^{aaa}<br>(-5.8; -3.0)<br>*n-56*
Amplitude of the TG-induced Ca^{2+} release ($\Delta F/F_0$)	**1.2**^{a,b,c,e}<br>(1.2; 1.2)<br>*n=42*	n/a

$^{aaa}p<0.001$, $^{aa}p<0.01$, $^{a}p<0.05$ *vs*. fresh oocytes
$^{bbb}p<0.001$, $^{bb}p<0.01$, $^{b}p<0.05$ *vs*. 9h *in vitro*
$^{ccc}p<0.001$, $^{cc}p<0.01$, $^{c}p<0.05$ *vs*. 25h *in vitro*
$^{ddd}p<0.001$, $^{dd}p<0.01$, $^{d}p<0.05$ *vs*. 9h *in vivo*
$^{eee}p<0.001$, $^{ee}p<0.01$, $^{e}p<0.05$ *vs*. 25h *in vivo*

Supplementary figure legends

Supplementary Fig. S1 Fertilization-induced Ca^{2+} oscillations in freshly ovulated and postovulatory aged oocytes

(A) Ca^{2+} trace representative for fresh oocytes. **(B)** Magnification of the 1st Ca^{2+} transient from **(A)** and a schematic representation of how the Ca^{2+} increase rate was calculated (Ca^{2+} decrease rate was calculated analogically). **(C-F)** Ca^{2+} traces representative for oocytes aged for 9 hrs *in vitro* **(C)**, 9 hrs *in vivo* **(D)**, 25 hrs *in vitro* **(E)** and 25 hrs *in vivo* **(F)**. In case of **(E-F),** two of the most frequently occurring types of Ca^{2+} oscillations were presented.

Supplementary Fig. S2 Dynamics of the 3rd fertilization-induced Ca^{2+} transient and IP3R1 and SERCA2 expression in postovulatory aged oocytes

(A-B) Point charts presenting the amplitude and the duration of the 3rd Ca^{2+} transient in freshly ovulated oocytes and oocytes aged for 9 and 25 hrs *in vitro* **(A)** and *in vivo* **(B)**. **(C-D)** Point charts presenting the rates of Ca^{2+} increase and decrease during the 3rd Ca^{2+} transient in freshly ovulated oocytes and oocytes aged for 9 and 25 hrs *in vitro* **(C)** and *in vivo* **(D)**. **(A-D)** Each dot represents one oocyte, the number of analysed oocytes is included in Table 1. **(E)** Original images of the Western blots showing expression of IP3R1 and SERCA2 proteins in brain lysate, freshly ovulated oocytes, oocytes aged for 9 and 25 hrs *in vitro* and *in vivo* and two other samples unrelated to this paper. The red line marks regions showed in Fig. 2 E as blots labelled for IP3R1 and SERCA2, blue line – a region showed in the Ponceau S staining.

Supplementary Fig. S3 Effect of cumulus cells on Ca^{2+} homeostasis in postovulatory aged oocytes.

(A) The mean Ca^{2+} release triggered by thapsigargin (TG), calculated for oocytes aged for 9 hrs *in vivo* and *in vitro* with and without cumulus cells (CC). The number of analysed oocytes is included in Tables 1 and 2. Mean values +/- SD are shown. Time-point "0" was set as a moment when the cytoplasmic Ca^{2+} concentration in oocytes started to rise. **(B)** Ca^{2+} trace representative for oocytes aged for 9 hrs *in vitro* with CC. **(C-G)** Point charts presenting the total duration of the Ca^{2+} oscillations and the mean interval between Ca^{2+} transients **(C)**, the amplitude and the duration of the 1st Ca^{2+} transient **(D)**, the amplitude and the duration of the 3rd Ca^{2+} transient **(E)**, the rates of Ca^{2+} increase and decrease during the 1st Ca^{2+} transient **(F)**, and the rates of Ca^{2+} increase and decrease during the 3rd Ca^{2+} transient **(G)** in oocytes aged for 9 hrs *in vivo* or *in vitro* with and without CC. Each dot represents one oocyte, the number of analysed oocytes is included in Tables 1 and Supplementary Table S2.

Supplementary Fig. S4 Effect of oxidative stress on Ca^{2+} ER store and fertilization-induced Ca^{2+} oscillations in oocytes

(A) The Ca^{2+} release triggered by thapsigargin (TG) and A23187 ionophore in representative freshly ovulated oocytes pre-treated with H_2O_2. Arrow indicates when TG or ionophore was added. **(B)** Ca^{2+} trace representative for fresh oocytes pre-treated with H_2O_2. **(C-D)** Point charts presenting the amplitude and the duration of the 3rd Ca^{2+} transient **(C)** and the rates of Ca^{2+} increase and decrease during the 3rd Ca^{2+} transient **(D)** in fresh oocytes, fresh oocytes pre-treated with H_2O_2 or oocytes aged for 25 hrs *in vitro*. **(E)** The mean Ca^{2+} release triggered by thapsigargin (TG), calculated for freshly ovulated oocytes or oocytes aged for 25 hrs *in vitro* with and without *N*-acetylcysteine (NAC). The number of analysed oocytes is included in Tables 1 and 2. Mean values +/- SD are shown. Time-point "0" was set as a moment when the cytoplasmic Ca^{2+} concentration in oocytes started to rise. **(F)** Ca^{2+} trace representative for oocytes aged for 25 hrs *in vitro* with NAC. **(G-H)** Point charts presenting the amplitude and the duration of the 3rd Ca^{2+} transient **(G)** and the rates of Ca^{2+} increase and decrease during the 3rd Ca^{2+} transient **(H)** in fresh oocytes and oocytes aged for 25 hrs *in vitro* with and without *N*-acetylcysteine (NAC). **(C-D, G-H)** Each dot represents one oocyte, the number of analysed oocytes is included in Tables 1 and 2.

Supplementary Fig. S5 Specificity of TMRE and MgGreen stainings

(A-B) Mg^{2+} (MgGreen in blue) and Ca^{2+} (Rhod-2 in orange) traces recorded for oocytes subjected to FCCP **(A)** or thapsigargin (TG) **(B)**. Arrows indicate when FCCP or TG was added. **(C)** Confocal image of a representative oocyte stained with MgGreen, displaying predominantly cytoplasmic localization of the dye. Although there are some signs of potential internalization of the dye (arrowheads), it does not form a pattern typical for ER cistern distribution (see **Fig. 4E**). Scale bar 50 µm.

Supplementary Fig. S6 Effect of postovulatory ageing on the readjustment of ATP production during Ca^{2+} oscillations.

(A-D) Mg^{2+} (MgGreen in blue) and Ca^{2+} (Rhod-2 in orange) oscillations in representative oocytes aged for 9 hrs *in vitro* **(A)** or *in vivo* **(B)** and for 25 hrs *in vitro* **(C)** or *in vivo* **(D).** The cytoplasmic concentration of free Mg^{2+} ions is inversely proportional to the ATP concentration. **(E)** A decrease in the Mg^{2+} concentration, indicative of an increase in the ATP concentration, during the 1st Ca^{2+} transient (value 'a' – value 'b' in **Fig. 4 C**) calculated for freshly ovulated oocytes (n=31), oocytes aged *in vitro* for 9 and 25 hrs (n=25 and 11, respectively) and oocytes aged *in vivo* for 9 and 25 hrs (n=18 and 32). $^{a}p<0.05$, $^{b}p<0.001$ *vs*. fresh oocytes. **(F)** A decrease in the Mg^{2+} concentration, indicative of an increase in the ATP concentration, during the 3rd Ca^{2+} transient (value 'c' – value 'd' in **Fig. 4 C**) calculated for freshly ovulated oocytes (n=31), oocytes aged *in vitro* for 9 and 25 hrs (n=25 and 10,

respectively) and oocytes aged *in vivo* for 9 and 25 hrs (n=15 and 29). [a]p<0.001, [b]p<0.01 *vs*. fresh oocytes, [c]p<0.001 *vs*. 9 hrs *in vitro*. **(A-B)** Graphs present medians and the 1st and the 3rd quartile values. The ends of the whiskers are set at 1.5*IQR above the third quartile and 1.5*IQR below the first quartile. Dots show the minimum and maximum values if they are outside the range (outliers).

Supplementary Fig. S7 Effect of actin depolymerisation on fertilization-induced Ca^{2+} oscillations in oocytes

(A) Ca^{2+} trace for a representative fresh oocyte treated with cytochalasin D (CCD). **(B-C)** Point charts presenting the amplitude and the duration of the 3rd Ca^{2+} transient **(B)** and the rates of Ca^{2+} increase and decrease during the 3rd Ca^{2+} transient **(C)** in freshly ovulated oocytes treated with CCD, and oocytes aged for 25 hrs *in vitro* and *in vivo*. Blue ovals indicate regions, where dots representing freshly ovulated oocytes would have been located. Each dot represents one oocyte, the number of analysed oocytes is included in Tables 1 and 2.

Supplementary Fig. S8 Effect of parthenogenetic activation with EtOH on fertilization-induced Ca^{2+} oscillations in oocytes

(A) Images of fresh oocytes, oocytes aged for 9 hrs *in vivo*, and fresh oocytes activated with ethanol (EtOH) or 6-DMAP and cycloheximide (CHX) used for the recording of Ca^{2+} oscillations. Images were taken just before fertilization. Asterisks indicate forming 2nd polar bodies and arrowheads – anaphase bulges. Scale bar 100 µm. **(B)** Ca^{2+} trace for a representative fresh oocyte pre-activated with EtOH. **(C-D)** Point chart presenting the amplitude and the duration of the 3rd Ca^{2+} transient **(C)** and the rates of Ca^{2+} increase and decrease during the 3rd Ca^{2+} transient **(D)** in freshly ovulated oocytes, oocytes pre-activated with EtOH and oocytes aged for 9 hrs *in vivo*. **(A-D)** Each dot represents one oocyte, numbers of analysed oocytes are included in Tables 1 and 2.

Supplementary Fig. S9 Effect of parthenogenetic activation with 6-DMAP and cycloheximide on fertilization-induced Ca^{2+} oscillations in oocytes

(A) Ca^{2+} trace for a representative fresh oocyte pre-activated with 6-DMAP and cycloheximide (CHX). **(B-F)** Point charts presenting the total duration of Ca^{2+} oscillations and the mean interval between Ca^{2+} transients **(B)**, the amplitude and the duration of the 1st Ca^{2+} transient **(C)**, the amplitude and the duration of the 3rd Ca^{2+} transient **(D)**, the rates of Ca^{2+} increase and decrease during the 1st Ca^{2+} transient **(E)**, and the rates of Ca^{2+} increase and decrease during the 3rd Ca^{2+} transient **(F)** in freshly ovulated oocytes, oocytes pre-activated with 6-DMAP and CHX and oocytes aged for 9 hrs *in vivo*. Each dot represents one oocyte, numbers of analysed oocytes are included in Tables 1 and Supplementary Table S2.

Supplementary Figure S1

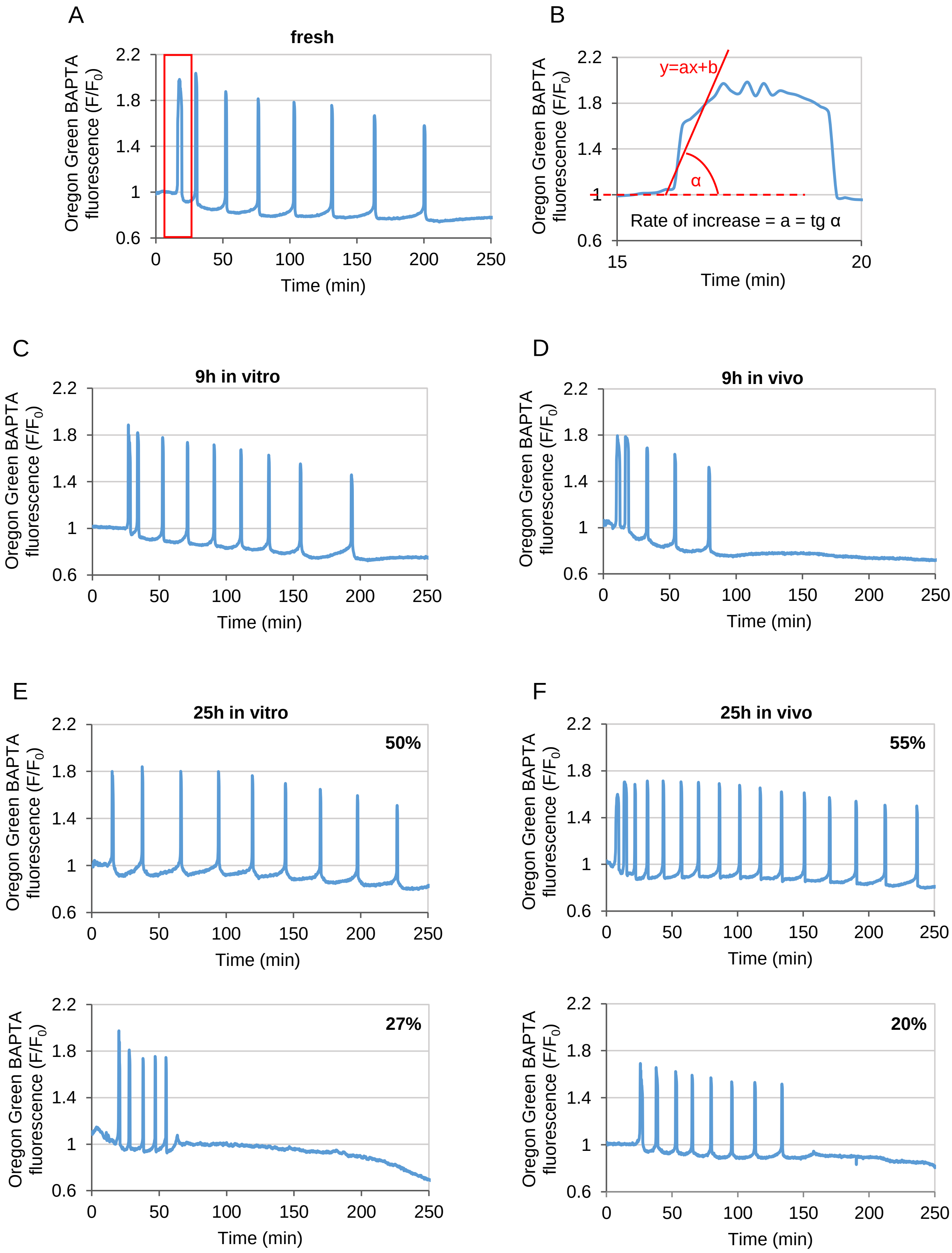

Supplementary Figure S2

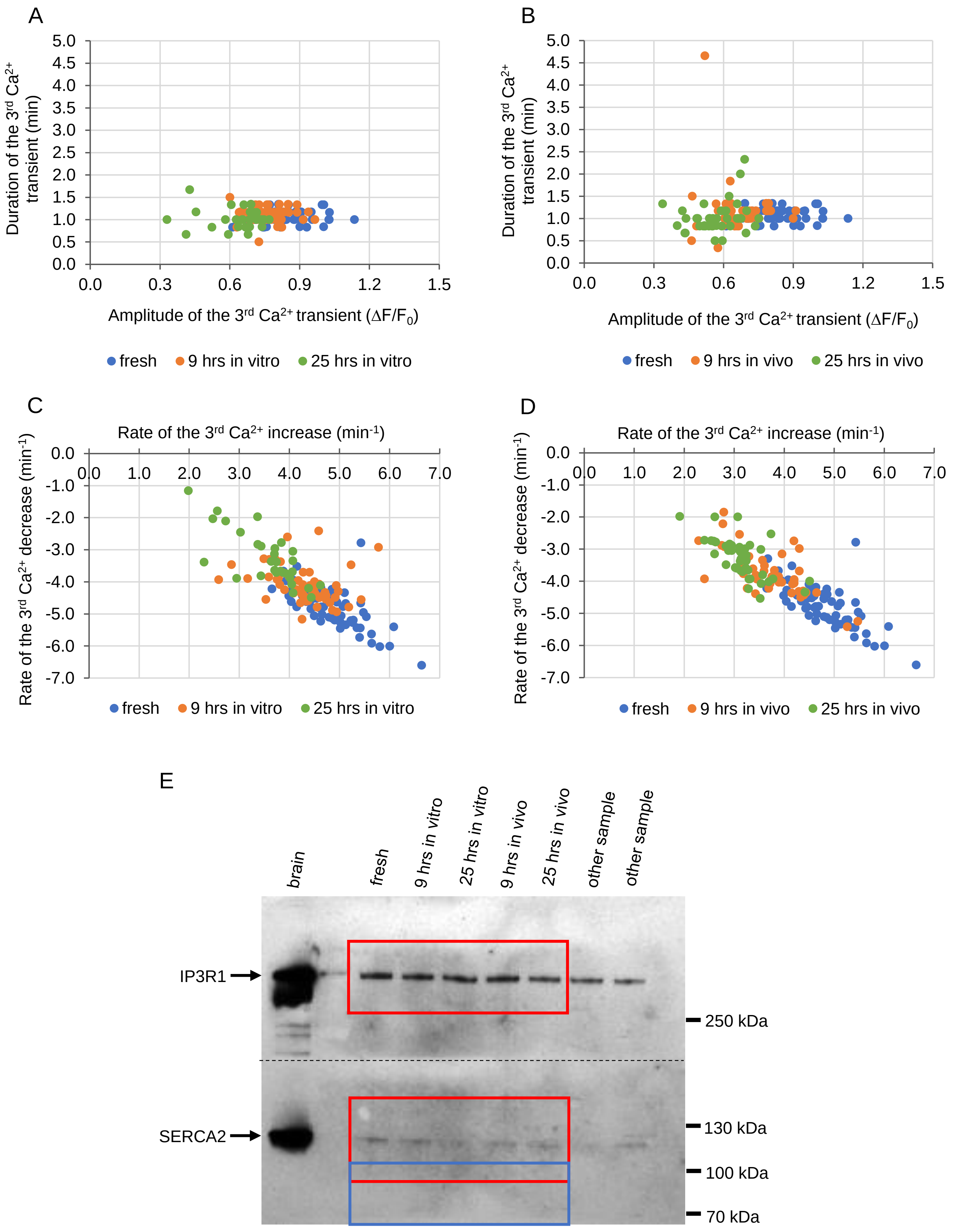

Supplementary Figure S3

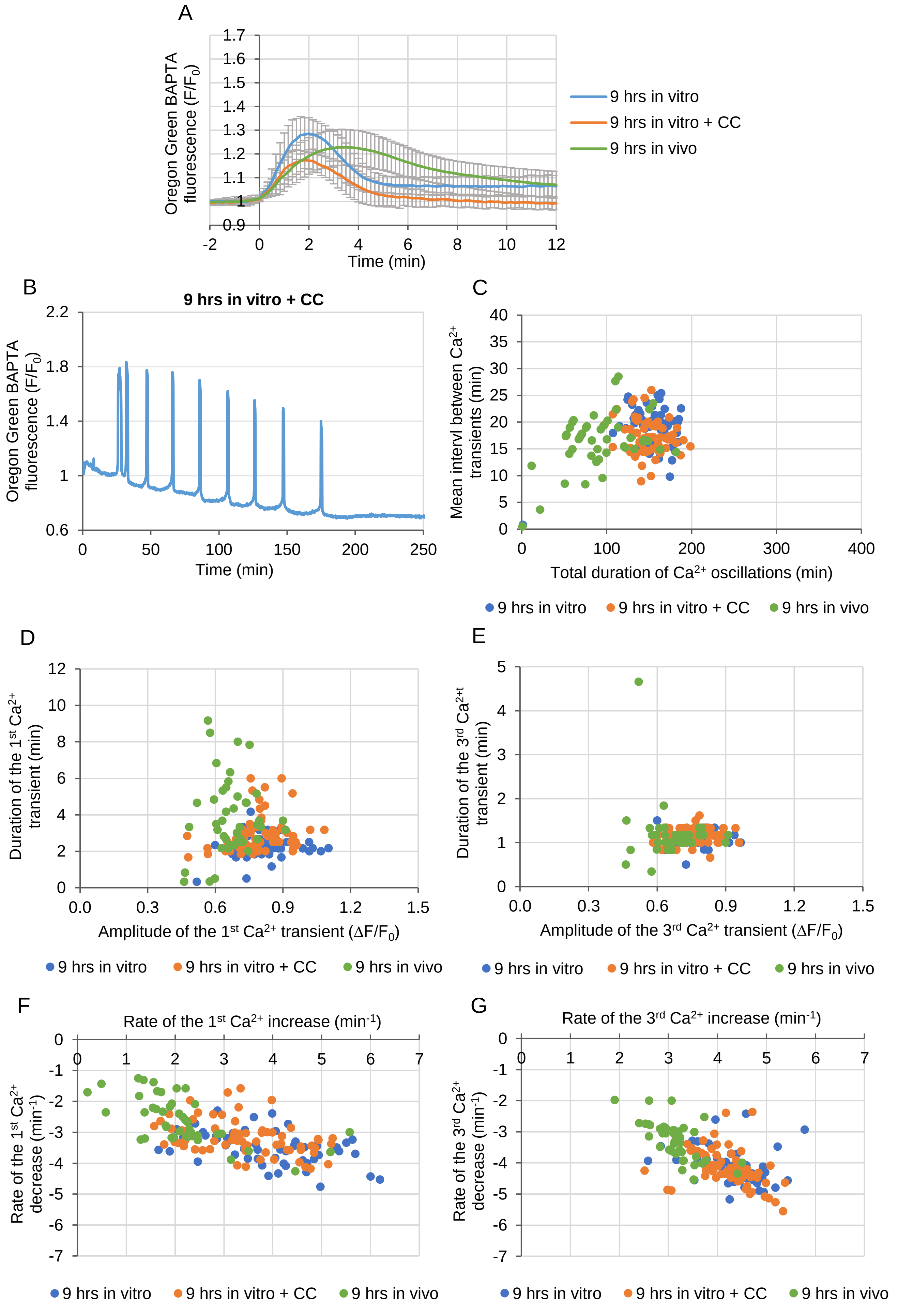

Supplementary Figure S4

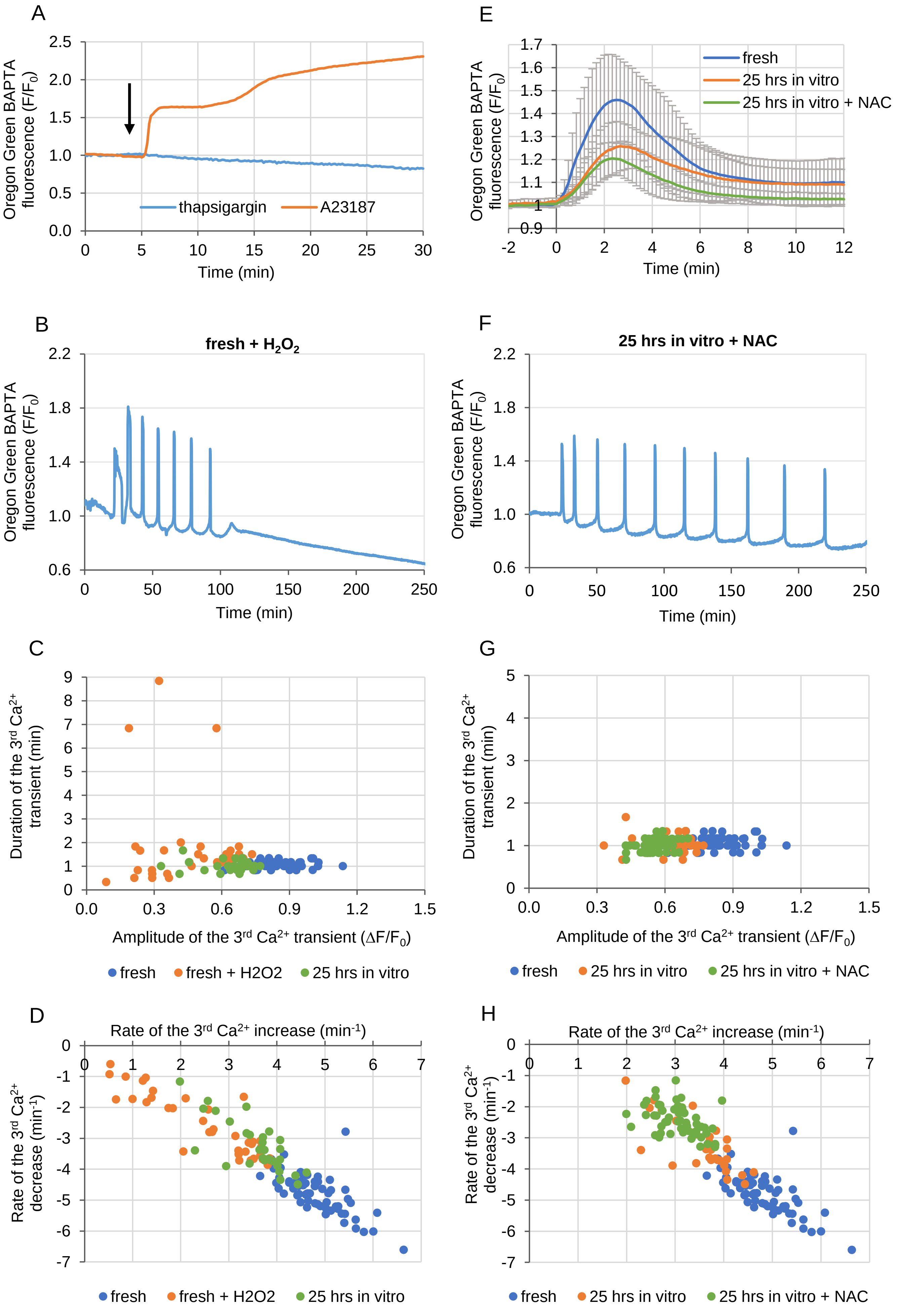

Supplementary Figure S5

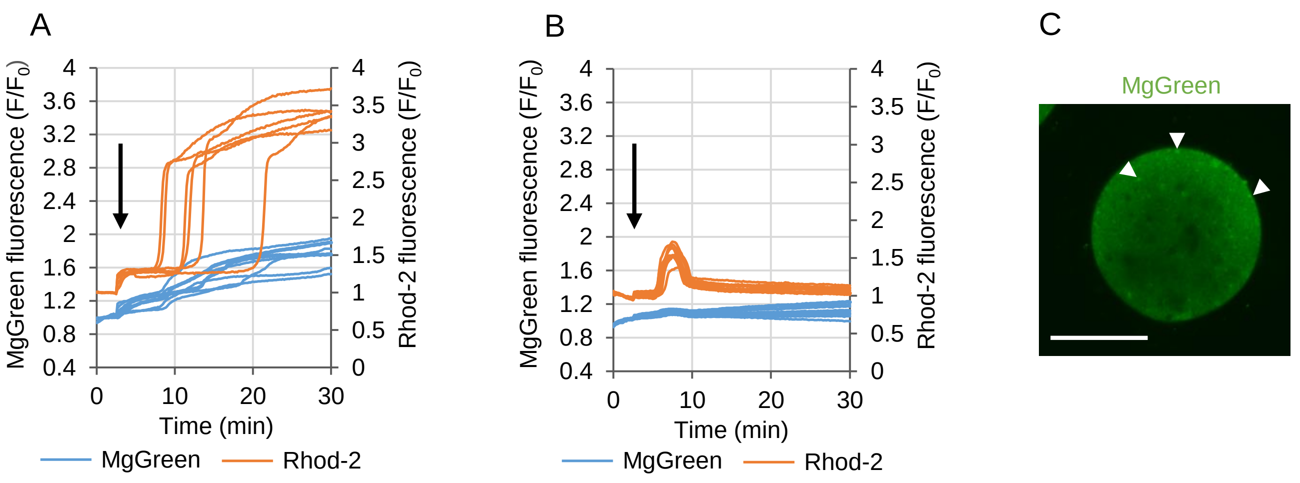

Supplementary Figure S6

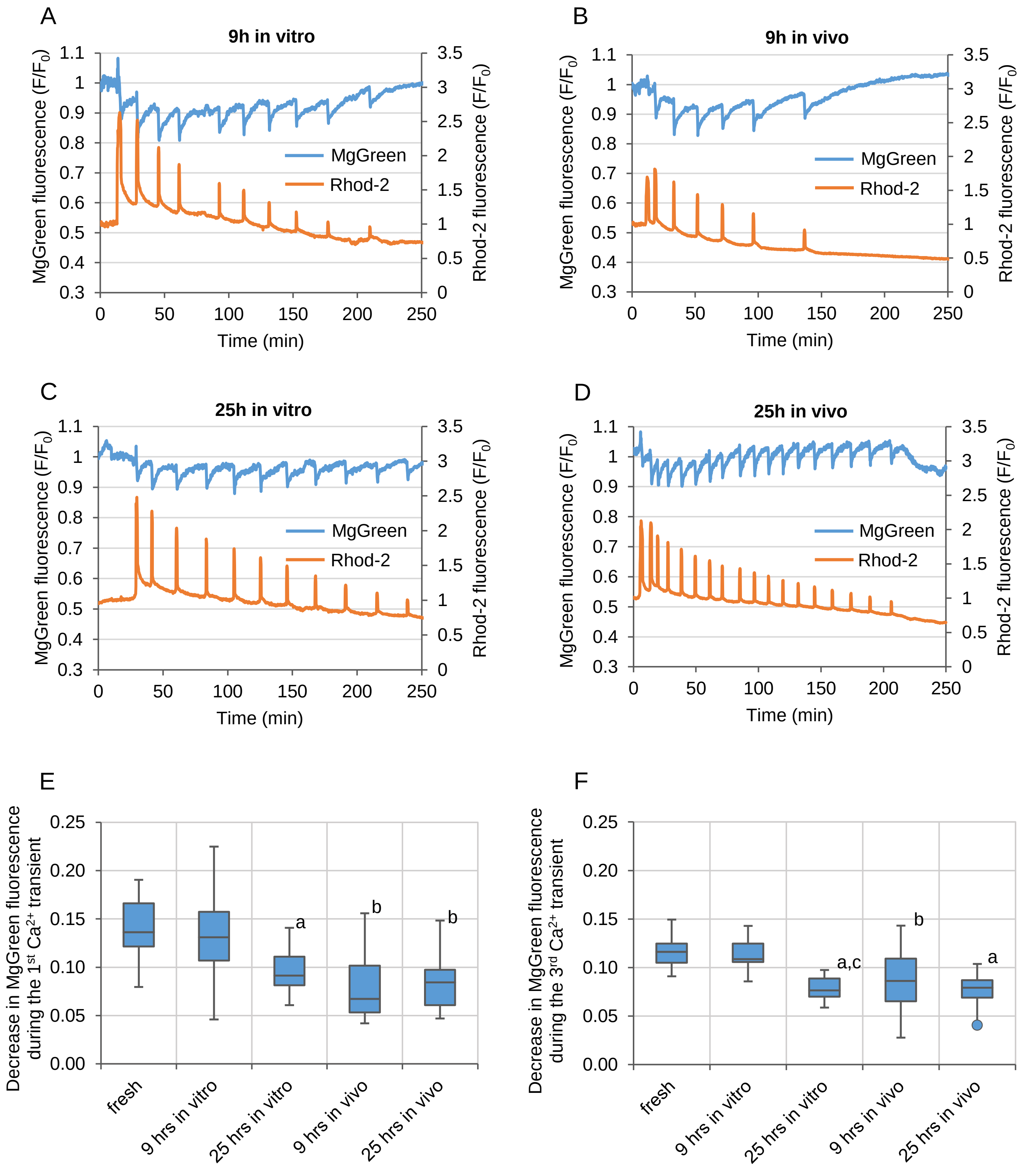

Supplementary Figure S7

A

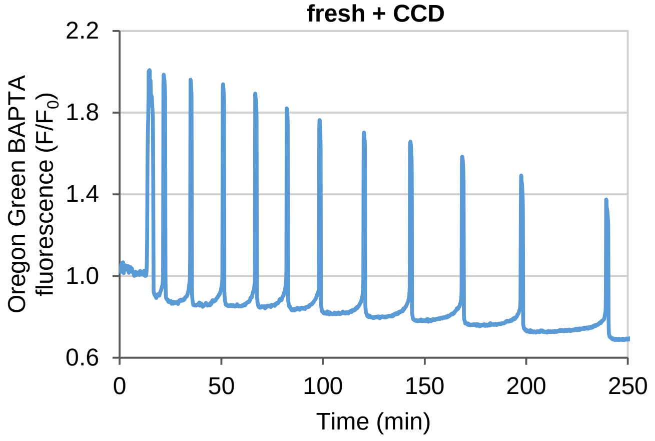

B

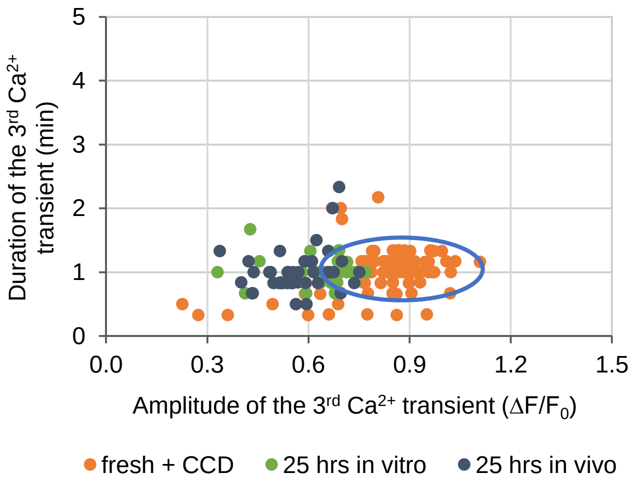

C

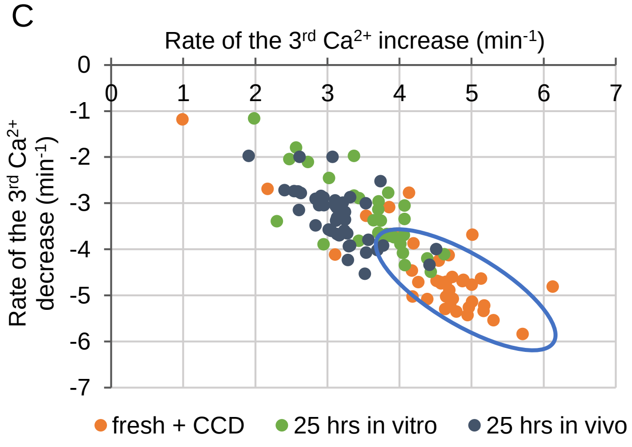

Supplementary Figure S8

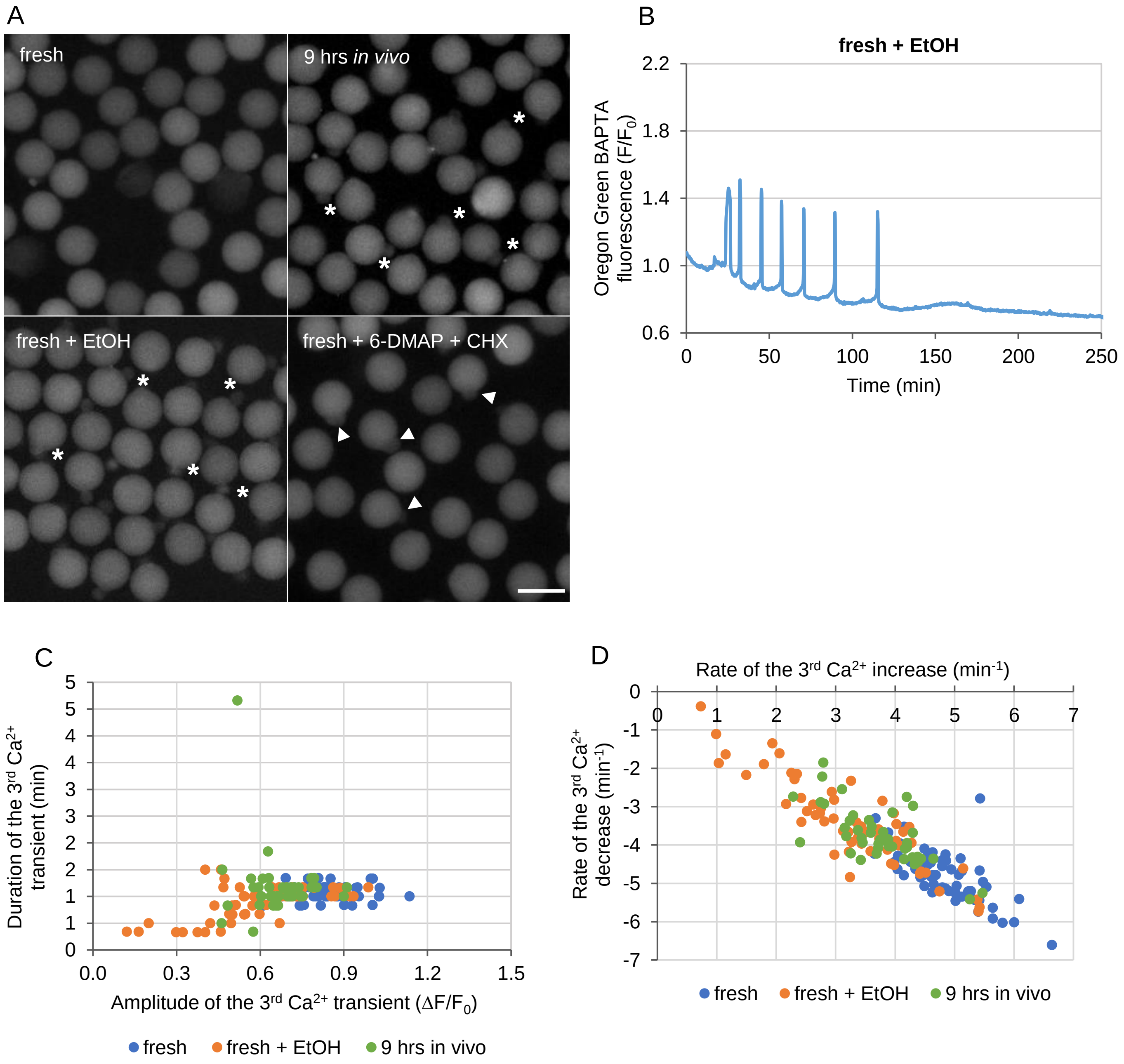

Supplementary Figure S9

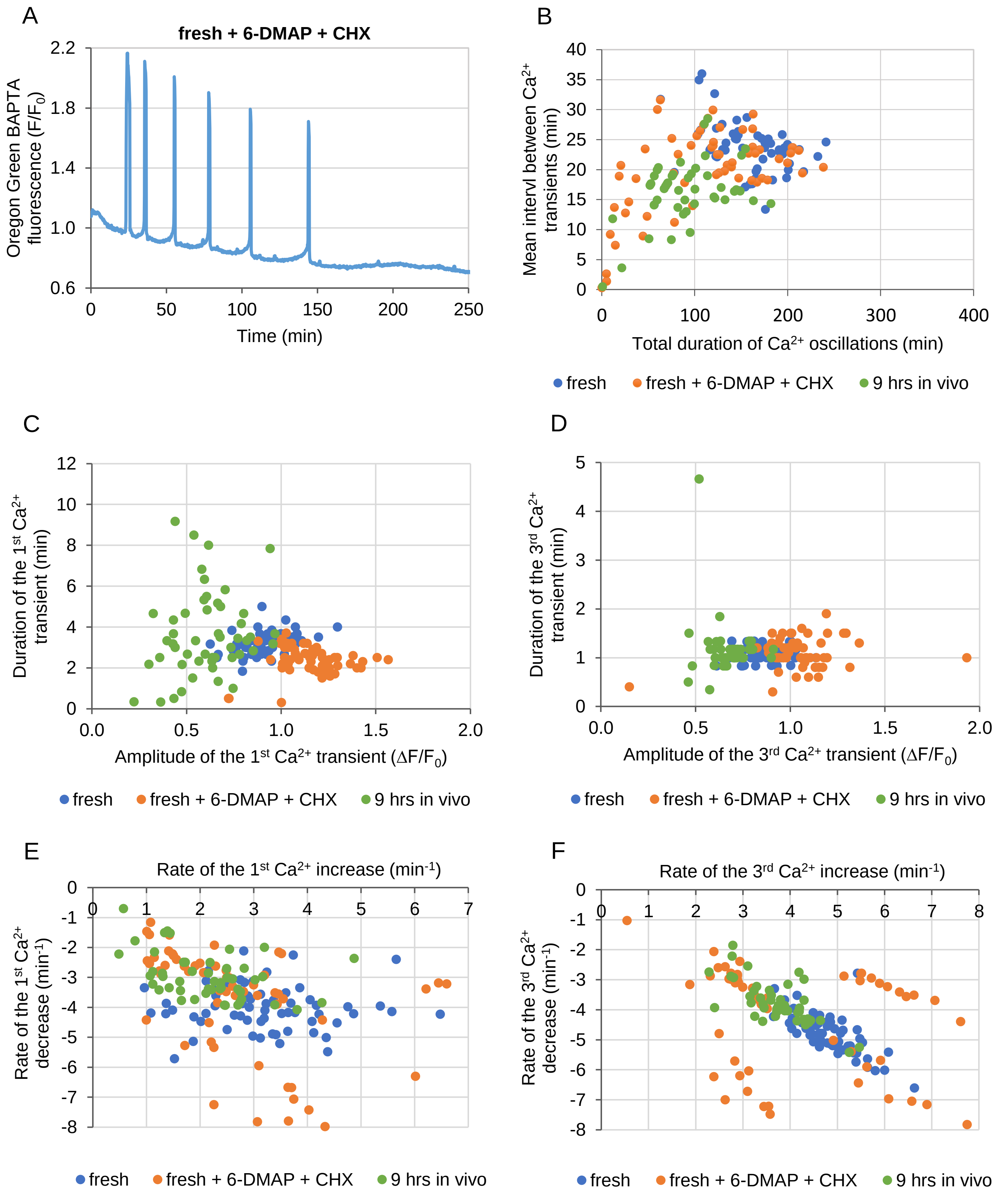

Supplement: Supplementary file 1 — Supplementary Information [file 41598_2019_48281_MOESM1_ESM.pdf]
